# Aluminum hydroxide adjuvant diverts the uptake and trafficking of genetically detoxified pertussis toxin to lysosomes in macrophages

**DOI:** 10.1111/mmi.14900

**Published:** 2022-04-07

**Authors:** Javier R. Jaldin‐Fincati, Serene Moussaoui, Maria Cecilia Gimenez, Cheuk Y. Ho, Charlene E. Lancaster, Roberto J. Botelho, Salvador F. Ausar, Roger H. Brookes, Mauricio R. Terebiznik

**Affiliations:** ^1^ Department of Biological Sciences University of Toronto at Scarborough Toronto Ontario Canada; ^2^ Department of Cell and Systems Biology University of Toronto at Scarborough Toronto Ontario Canada; ^3^ Department of Chemistry and Biology Ryerson University Toronto Ontario Canada; ^4^ BioProcess Research and Development Sanofi Pasteur Ltd. Toronto Ontario Canada; ^5^ Present address: Instituto de Patología Experimental CONICET—Universidad Nacional de Salta Salta Argentina

**Keywords:** aluminum hydroxide adjuvant, genetically detoxified pertussis toxin, macrophages, macropinocytosis, phagocytosis

## Abstract

Aluminum salts have been successfully utilized as adjuvants to enhance the immunogenicity of vaccine antigens since the 1930s. However, the cellular mechanisms behind the immune adjuvanticity effect of these materials in antigen‐presenting cells are poorly understood. In this study, we investigated the uptake and trafficking of aluminum oxy‐hydroxide (AlOOH), in RAW 264.7 murine and U‐937 human macrophages‐like cells. Furthermore, we determined the impact that the adsorption to AlOOH particulates has on the trafficking of a *Bordetella pertussis* vaccine candidate, the genetically detoxified pertussis toxin (gdPT). Our results indicate that macrophages internalize AlOOH by constitutive macropinocytosis assisted by the filopodial protrusions that capture the adjuvant particles. Moreover, we show that AlOOH has the capacity to nonspecifically adsorb IgG, engaging opsonic phagocytosis, which is a feature that may allow for more effective capture and uptake of adjuvant particles by antigen‐presenting cells (APCs) at the site of vaccine administration. We found that AlOOH traffics to endolysosomal compartments that hold degradative properties. Importantly, while we show that gdPT escapes degradative endolysosomes and traffics toward the retrograde pathway, as reported for the wild‐type pertussis toxin, the adsorption to AlOOH diverts gdPT to traffic to the adjuvant’s lysosome‐type compartments, which may be key for MHC‐II‐driven antigen presentation and activation of CD4^+^ T cell. Thus, our findings establish a direct link between antigen adsorption to AlOOH and the intracellular trafficking of antigens within antigen‐presenting cells and bring to light a new potential mechanism for aluminum adjuvancy. Moreover, the in‐vitro single‐cell approach described herein provides a general framework and tools for understanding critical attributes of other vaccine formulations.

## Graphical abstract

Our findings establish a direct link between antigen adsorption to aluminum hydroxide (AlOOH) and the intracellular trafficking of antigens within macrophages, bringing to light a potential simple mechanism for aluminum adjuvancy. The genetically detoxified pertussis toxin (gdPT) antigen model escapes degradative endolysosomes and traffics toward the retrograde pathway. However, adsorption to AlOOH particles diverts gdPT to traffic to the adjuvant’s endolysosomal compartment. AlOOH‐adsorbed antigens may be key for MHC‐II‐driven antigen presentation.

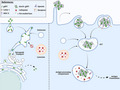

## 1 INTRODUCTION

Aluminum salts form microparticles of crystalline or amorphous nature that have been used as adjuvants to enhance the immunogenicity of diverse vaccine formulations with outstanding results for several decades now. Potassium aluminum sulfate was the first compound to be shown to boost immunogenicity, utilizing diphtheria toxoid in guinea pigs (Glenny et al., 1926). Currently, aluminum phosphate and aluminum hydroxide are the most used adjuvants in licensed human vaccines. These preformed aluminum salts present several practical advantages over outdated in situ antigen precipitation with potassium aluminum sulphate. This includes the possibility of establishing more standardized preparation protocols, the ability to capture antigens by direct adsorption instead of precipitation, and the improvement of the adsorption/elution performance of vaccines in vivo (Hem & Hogenesch, 2007b). Moreover, current aluminum‐based adjuvants are used in combination with microbial‐recognition receptors agonists to enhance immunogenicity (Guy, 2007).

Although the antigen adsorption onto aluminum‐based adjuvants is mainly mediated by electrostatic and ligand exchange interactions at the surface and crevices of the adjuvant microparticles, other attractive forces may contribute to this process, such as hydrogen bonding, hydrophobic interactions, and van der Waals forces (Hem & Hogenesch, 2007a; Peek et al., 2007). In addition, antigens can be simply entrapped in void spaces within particle aggregates during vaccine preparation, favoring their uniform distribution (Hem & Hogenesch, 2007b; Romero Mendez et al., 2007).

The antigen entrapment and adsorption capacities of aluminum preparations are crucial for their immunopotentiation effect (Hem et al., 2010; Hogenesch, 2012), as changing antigens from soluble to a particulate state may slow down the diffusion of antigens in the site of vaccine administration, a mechanism known as depot effect (Awate et al., 2013), and hence, facilitate their capture and internalization by antigen‐presenting cells (APCs) (Hogenesch, 2012; Morefield et al., 2005). Within APCs, the internalized antigens traffic to hydrolytic phagosomes and endolysosomal compartments where peptides are produced and loaded onto major histocompatibility complex type II (MHC‐II) receptors for antigen presentation and activation of CD4+ T cells (Ghimire et al., 2012). However, the immunopotentiation effect of aluminum adjuvants has also been associated with the recruitment and activation of APCs at the site of vaccine administration following strong inflammatory stimuli (Awate et al., 2013). This phenomenon leans on aluminum adjuvants’ ability to cause oxidative damage of endosomal membranes and the activation of the NLRP3 inflammasome, and consequently, proinflammatory downstream signaling cascades, caused by hydroxyl radicals (Eisenbarth et al., 2008; Hogenesch, 2012; Mold et al., 2016; Reinke et al., 2020). Although broadly accepted, this mechanism for aluminum adjuvancy remains controversial since NLRP3 independent mechanisms have also been reported (Franchi & Nunez, 2008; Marrack et al., 2009; McKee et al., 2009). Moreover, how these two opposing mechanisms, one requiring functional endosomes for antigen presentation, and the other leaning on damaged compartments, concur in an immunopotentiation effect is currently unknown. In fact, the uptake and intracellular trafficking of aluminum adjuvant particles are poorly understood phenomena, despite being central to the adjuvating function of these compounds. Furthermore, it is unknown if aluminum adjuvants can influence the trafficking of antigens such as toxins and viral proteins. These are co‐administered in vaccine formulations and antigens are often treated to neutralize their toxicity by either chemical or genetic means, yet it remains possible that some of these proteins may still preserve the ability to escape the endocytic pathway in a mechanism encoded in their molecular structure (do Vale et al., 2016; Johannes & Decaudin, 2005). This is the case of the genetically detoxified mutant of pertussis toxin (gdPT). Pertussis toxin (PTx) is an AB_5_ exotoxin produced by *Bordetella pertussis*, the etiological agent of the acute respiratory infection known as whoopping cough. Upon entering the cell via clathrin‐mediated endocytosis, PTx escapes the endocytic pathway to traffic retrogradely to the Golgi apparatus and the endoplasmic reticulum (ER) (el Baya et al., 1997; Plaut & Carbonetti, 2008). It has been proposed that from the ER, the enzymatic A‐domain of PTx translocates into the cytoplasm to exert its toxic effect (Banerjee et al., 2016; Locht et al., 2011; Roy et al., 2006). The S1 subunit that forms the A‐domain catalyzes the ribosylation of heterotrimeric inhibitory Gi proteins, leading to the increase in cAMP cellular levels and the disruption of signaling by different G protein‐coupled receptors (Hsia et al., 1985; Kugler et al., 2007; Locht et al., 2011; Tamura et al., 1983). The genetically detoxified variant of PTx (gdPT) was developed by introducing a double point mutation (R9K/E129G) in the S1 subunit of PTx A domain (Burnette et al., 1988; Dewan et al., 2020; Pizza et al., 1989). These mutations substantially reduced the enzymatic activity of gdTP and completely abolished its toxicity, albeit holding a near‐identical structure to that of the wild‐type toxin (Ausar et al., 2020; Gregg & Merkel, 2019; Loosmore et al., 1990; Seubert et al., 2014).

Herein, we investigated the cellular uptake and intracellular trafficking of aluminum hydroxide (AlOOH) particles in the RAW 264.7 murine and in the U‐937 human macrophage‐like cells, and how the adsorption to adjuvant particles affects the intracellular fate of gdPT. Our results show that AlOOH particles are readily internalized by constitutive macropinocytosis, which is characteristic of APCs (Doodnauth et al., 2019; Norbury, 2006). Moreover, the ability of AlOOH to nonspecifically adsorb IgG enhanced the cellular uptake of the adjuvant particles by engaging phagocytosis, which may more closely reflect the fate of AlOOH particles at the site of vaccine administration. Importantly, we found that most of the internalized AlOOH particles traffic to intracellular compartments endowed with endolysosomal degradative properties. Furthermore, we show that while AlOOH‐absorbed gdPT traffics with the adjuvant particles to these degradative endosomal compartments, most of the internalized unadjuvanted gdPT localizes to abnormal endosomes that lack hydrolytic activity. Altogether, our findings indicate that the adsorption to aluminum adjuvants may divert antigens from their typical trafficking route toward endolysosomal compartments, therefore bringing to light a simple mechanism for aluminum adjuvanticity.

## 2 RESULTS

### 2.1 Capture and uptake of AlOOH particles by macrophages

**FIGURE 1:**
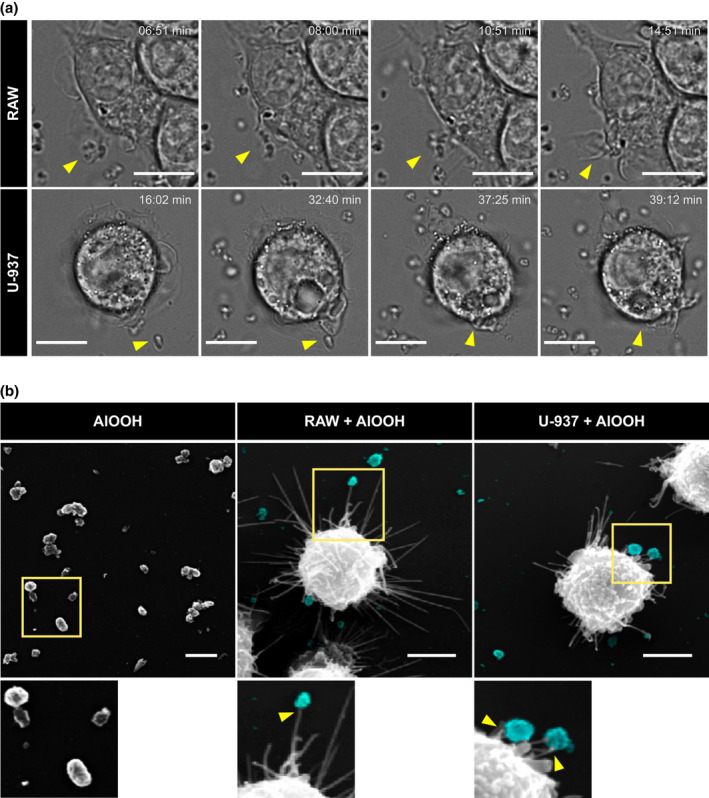
Uptake of aluminum adjuvant particles by macrophages. (a) RAW or U‐937 cells were incubated with AlOOH for 5 min at 37°C prior to live cell bright field microscopy. Representative stills showing the capture and internalization of adjuvant particles over time are depicted and individual internalization events highlighted with yellow arrowheads. (b) RAW or U‐937 cells were incubated with AlOOH as described above and at 30 min of internalization, cells were processed for SEM. Representative SEM micrographs displaying macrophage filopodial protrusions interacting with aluminum adjuvant (pseudocolor cyan) are shown in middle and right panels (yellow arrowheads). Images are representative of three independent trials. Fifty cells per trial per condition were analyzed. Scale bars, 5 μm

**FIGURE 2:**
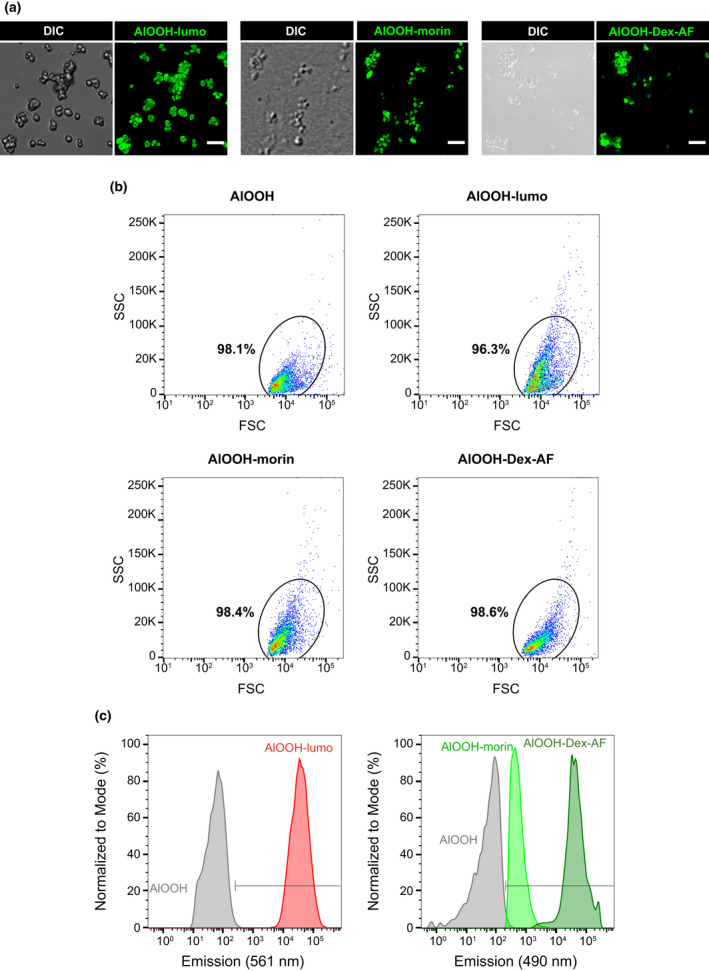
Fluorescent labeling of aluminum adjuvant particles. (a) AlOOH preparations were conjugated with lumogallion, morin or dextran conjugated with Alexa Fluor® dye overnight at 4°C prior to visualization by spinning disk confocal microscopy. Representative micrographs showing each fluorophore‐adjuvant conjugate by differential interference contrast (DIC) and fluorescent microscopy are depicted. (b–c) Fluorophore‐AlOOH conjugates were processed by flow cytometry. Representative scatterplots showing the labeling efficiency for AlOOH‐lumo, AlOOH‐morin, and AlOOH‐Dex‐AF are shown in (b). The mean fluorescence intensity profiles for AlOOH‐lumo (em: 561 nm), AlOOH‐morin (em: 490 nm), and AlOOH‐Dex‐AF (em: 491 nm) from the selected regions in (b) are shown in histograms depicted in (c). Spinning disk confocal images are a merge of z‐stacks. Images are representative of three independent trials. Scale bars, 5 μm

**FIGURE 3:**
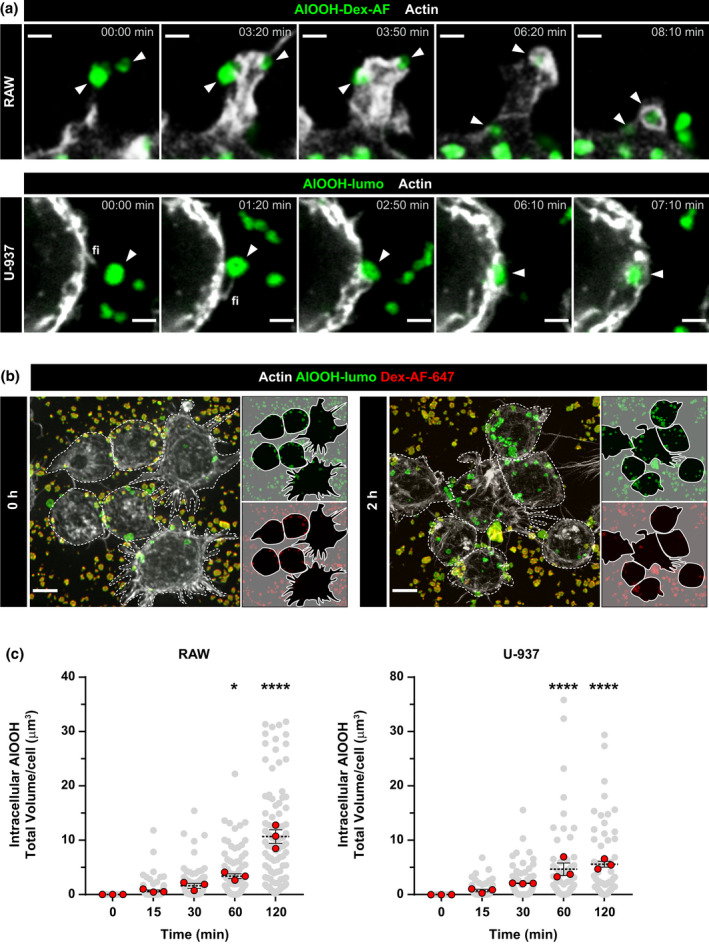
Uptake of fluorescent aluminum adjuvant and quantitative analysis. (a) RAW cells stably transfected with life‐act‐RFP or U‐937 cells pretreated with SiR‐Actin probe were incubated with dextran‐AF or lumo‐conjugated AlOOH, respectively, for 5 min at 37°C prior to live cell imaging. Representative stills from Video S3 showing internalization of AlOOH‐Dex‐AF488 or AlOOH‐lumo are depicted. White arrowheads point to aluminum adjuvant particles undergoing internalization. “Fi” labels macrophage filipodial protrusions. (b) RAW cells were incubated with AlOOH‐lumo for 0 or 2 h at 37°C and subsequently processed for differential labeling of internal/external aluminum adjuvant particles, as described in experimental procedures. Representative confocal micrographs show internal versus external adjuvants associated with RAW cells. To allow for cell delineation, F‐Actin was stained with blue phalloidin (pseudocolored gray). Cells associated with adjuvant particles either attached (green and red) or internalized (green) are outlined with white dotted lines. (c) Volocity® quantifications of the total volume (μm^3^) of AlOOH‐lumo particles internalized per cell over time, in both RAW and U‐937 cells, are represented in the scatter plots, where each red dot corresponds to the mean of an independent experiment and each dashed line represents the average ± SEM. *p ≤ .05, ****p ≤ .0001. Spinning disk confocal images represent a merge of z‐stacks. Images are representative of three independent trials. Fifty cells per trial per condition were analyzed. Scale bars, 5 μm

To investigate the interaction of AlOOH with macrophages, we first characterized the capture and uptake of antigen‐free adjuvant particles by RAW 264.7 murine and U‐937 human macrophage‐like cells (hereinafter referred to as RAW and U‐937 macrophages, respectively) by live cell microscopy. As shown in Videos S1 and S2, RAW and U‐937 macrophages undergo continuous membrane ruffling activity and cast filopodial protrusions that scout the cellular periphery. These protrusions drag the AlOOH particles toward the ruffling membranes where the cells swallow them. Figure 1a shows still‐images from Videos S1 and S2 that exemplify this process. The SEM micrographs from Figure 1b show macrophage filopodial protrusions contacting AlOOH particles at a higher resolution. To distinguish AlOOH particles from cell‐borne confounders during internalization assays, we resorted to labeling AlOOH for fluorescence microscopy. To this end, we tested labeling AlOOH with the fluorescent compounds lumogallion, morin hydrate, and dextran conjugated with Alexa Fluor^Ⓡ^ dyes. Lumogallion is an aluminum chelator that forms a fluorescent complex with AlOOH (AlOOH‐lumo) that can be excited at 490 nm and displays a wide emission spectrum (520–650 nm) that peaks at 580 nm (Mile et al., 2015). Morin forms a highly fluorescent complex with AlOOH (AlOOH‐morin) that has its maximum excitation and emission peaks at 418 and 490 nm, respectively (Mile et al., 2015). As an alternative to these methods, we labeled AlOOH particles with Alexa Fluor^Ⓡ^ fluorescent dyes conjugated to the anionic polysaccharide dextran, which have narrower excitation‐emission peaks than lumogallion and morin and are available for different fluorescent emissions. Thus, Alexa Fluor® dyes present the technical advantage of being highly combinable with other fluorophores used in fluorescence microscopy. Overnight incubation at room temperature of AlOOH with either dextran‐Alexa Fluor 488, lumogallion, or morin resulted in a highly efficient staining of the adjuvant particles through the formation of stable fluorescent complexes, detectable by either microscopy or flow cytometry analysis (Figure 2a–c). Next, utilizing fluorescently labeled AlOOH particles, we tracked the internalization of adjuvant particles by RAW and U‐937 macrophages by confocal live‐cell microscopy. As shown in Figure 3a and Videos S3 and S4, AlOOH particles were engulfed via two processes. First, uptake of AlOOH particles occurred via filopodial‐like structures that seized and dragged the particles toward the cell. Second, AlOOH particles were engulfed at areas of the cell surface that underwent continuous ruffling, a process that requires approximately 6–7 min for completion (Videos S3 and S4 and Figure 3a). Whereas the former process resembles macrophages filopodia capturing bacteria and other particles via phagocytosis (Flannagan et al., 2010; Horsthemke et al., 2017; Moller et al., 2013); the latter concurs better with the ruffling activity associated with constitutive macropinocytosis, characteristic of APCs (Doodnauth et al., 2019). To distinguish whether macropinocytosis and/or phagocytosis were responsible for the uptake of AlOOH particles by macrophages, we treated cells with pharmacological inhibitory compounds with different specificities toward these pathways. To this end, we first implemented a fluorescent labeling method to distinguish between intracellular and extracellular AlOOH particles. Briefly, macrophages were allowed to internalize AlOOH‐lumo particles and at the time points required for the experimental design, the membrane‐bound extracellular AlOOH‐lumo particles were labeled with Alexa Fluor‐conjugated dextran, which is membrane impermeant (refer to experimental procedures for more details). As a result of this procedure, the extracellular AlOOH particles became easily distinguishable from those already internalized by the cell (Figure 3b). This procedure allowed the identification and quantification of intracellular particles by computer analysis obtained from synchronized internalization assays (see experimental procedures), as depicted in Figure 3b,c. For this analysis, the total volume of internalized AlOOH was plotted, instead of particle number, to account for the heterogenicity in the size of AlOOH particles (Figure S1). The results from Figure 3c shows that the amount of AlOOH internalized by the cells increased over time, which agrees with a process where macrophages capture and ingest particles laying at the cell’s surroundings, as could be observed in Videos [Link], [Link].

**FIGURE 4:**
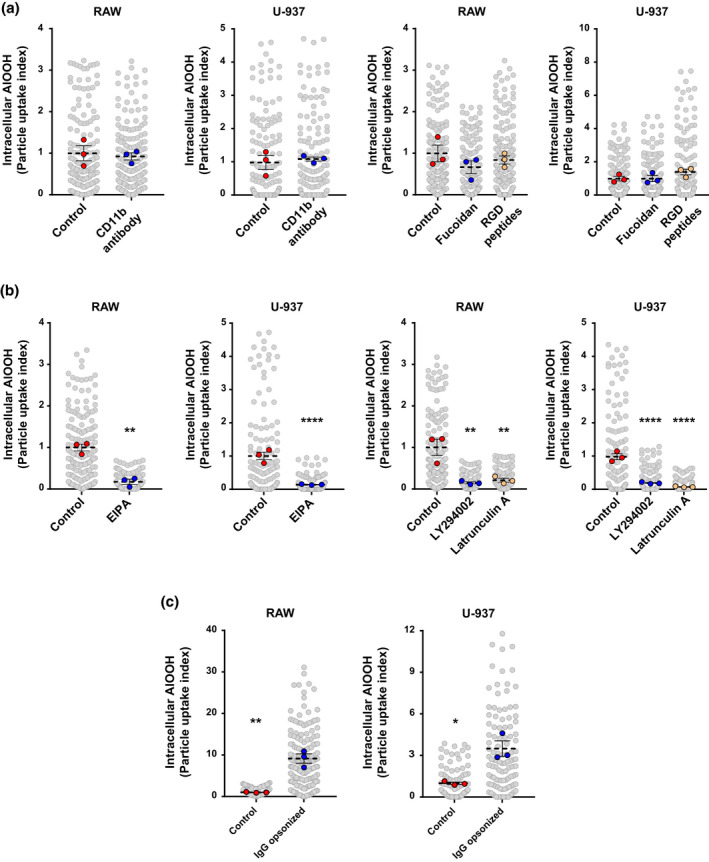
Aluminum adjuvant particles are internalized via macropinocytosis. (a and b) RAW or U‐937 cells were incubated with AlOOH‐lumo for 2 h at 37°C in the presence of the following inhibitory molecules or controls, respectively: CD11b blocking antibody (0.01 mg/ml), fucoidan (100 μg/ml), RGD peptides (0.1 mg/ml), EIPA (100 μM), LY294002 (50 μM), Latrunculin A (0.25 μM), IgG isotype control, DMEM media, methanol or DMSO. Subsequently, cells were processed for differential labeling of internal/external aluminum adjuvant particles. Scatter plots show the particle uptake index (refer to experimental procedures for details), for each treatment. **p ≤ .01, ****p ≤ .0001. (c) RAW or U‐937 cells were incubated with AlOOH‐lumo (control) or previously opsonized with human IgG for 2 h at RT, and subsequently processed for differential labeling of internal/external aluminum adjuvant particles. The particle uptake index for each condition was calculated as described above and represented in the scatter plots. One hundred cells for each condition in each of the three independent experiments were analyzed. *p ≤ .05, **p ≤ .01

We then determined the effect of different inhibitory treatments for macropinocytosis or phagocytosis on the internalization of AlOOH particles. First, we investigated the involvement of integrin and scavenger receptors in the internalization of AlOOH by macrophages, which have broad ligand specificity (Uribe‐Querol & Rosales, 2020). As shown in Figure 4a, neither inhibiting integrins, with RGD peptides, nor scavenger receptors, with fucoidan, blocked the uptake of AlOOH by macrophages (Hamasaki et al., 2018; O’Brien & Melville, 2003; Thelen et al., 2010). Furthermore, the internalization of AlOOH was not affected by blocking complement receptor 3 (CD11b/CD18), involved in opsonin and non‐opsonic phagocytosis (Gordon & Rice, 1988; Patel & Harrison, 2008). Although the uptake of AlOOH was not inhibited by blocking phagocytic receptors, the process was strongly inhibited by treating macrophages with the actin depolymerizing compound Latrunculin A, which was shown to inhibit both phagocytosis and macropinocytosis (Canton et al., 2016; Fujiwara et al., 2018) (Figure 4b). Similarly, AlOOH internalization was also inhibited by the phosphoinositide 3‐kinase inhibitor, LY294002, which blocks the production of phosphatidylinositol 3,4,5‐trisphosphate, a membrane signaling lipid that controls actin polymerization at the plasma membrane (Figure 4b). LY294002 inhibits macropinocytosis and phagocytosis of large particles (>3 μm) (Araki et al., 1996; Schlam et al., 2015). Moreover, the uptake of AlOOH was strongly inhibited by EIPA, a macropinocytosis‐specific inhibitor that prevents the activity of the membrane Na^+^/H^+^ exchanger 1, which is required for the activation of the small Rho GTPases Rac1 and Cdc42 during macropinocytosis (Koivusalo et al., 2010; Lin et al., 2018) (Figure 4b). Thus, collectively our results indicate that the uptake of AlOOH particles depend on macropinocytosis. However, unlike macropinocytosis of fluid‐phase solutes, our observations indicate that macropinocytosis of AlOOH requires filopodial protrusions that bind and drag the particles to the cell surface where macropinocytic events take place. Since none of the treatments applied to block receptors influenced AlOOH uptake efficiency, the binding of AlOOH to macrophage’s protrusions may be mediated by the capacity of the adjuvant particles to establish nonspecific binding interactions with multiple different molecular moieties expressed on macrophage surfaces (Flach et al., 2011).

Considering this, we reasoned that opsonic ligand may bind AlOOH in a nonselective fashion, thereby triggering their phagocytosis at the site of vaccine administration. To investigate this hypothesis, and because IgG is the principal immunoglobulin isotype found in interstitial fluids (Janeway et al., 2001), we assessed the effect of adsorbing nonspecific IgG to AlOOH on the uptake of AlOOH particles. As shown in Video S5, when RAW macrophages were confronted simultaneously with IgG‐adsorbed and naïve AlOOH particles, the former were captured at higher rates than the latter and via prominent phagocytic cups. Accordingly, the quantitative data from Figure 4c show that treating AlOOH with IgG significantly increased the uptake of adjuvant in both macrophage cell types. Altogether our observations indicate that although AlOOH particles are internalized by macropinocytosis, at the site of vaccine administration the nonspecific adsorption of IgG to AlOOH may favor a more efficient uptake of the adjuvant particles by APCs via phagocytosis, a phenomenon that could also apply to C3b and other opsonins present in interstitial fluids.

### 2.2 Impact of AlOOH particle size on its uptake

**FIGURE 5:**
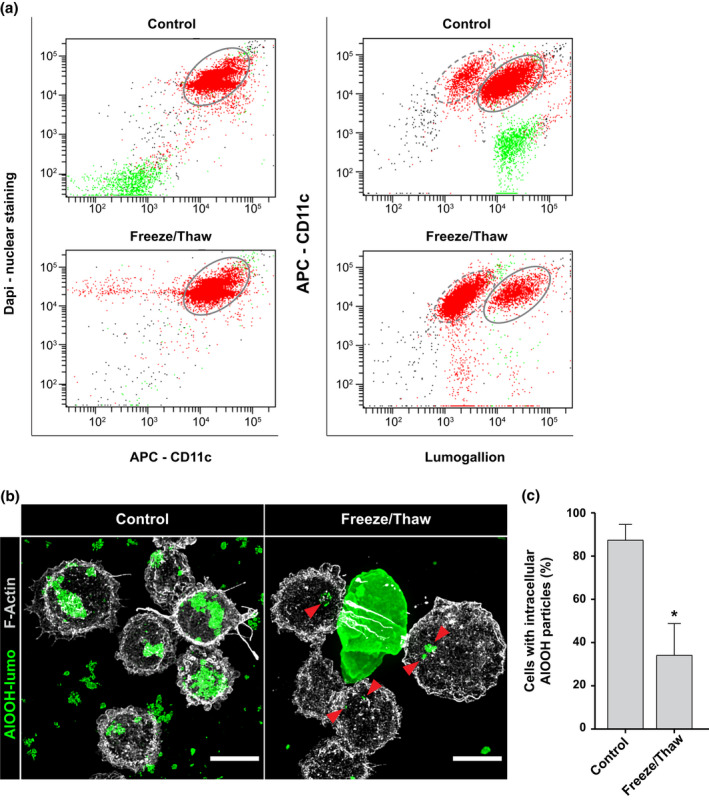
Impact of particle size on aluminum adjuvant internalization. (a) U‐937 cells were incubated with AlOOH‐lumo (control) or AlOOH‐lumo previously subjected to freeze/thaw cycles (freeze/thaw) to increase the size of the particles, as described in experimental procedures. The cells were incubated at 37°C for 4 h and subsequently processed for flow cytometry analysis. Differentiated macrophages were classified using anti‐CD11c antibodies. Cell nuclei were labeled with DAPI. Cell populations encircled by a dotted line have not internalized AlOOH‐Lumo and cells that have internalized AlOOH‐Lumo are encircled by a solid line. (b) U‐937 cells were incubated with control or freeze/thaw AlOOH‐lumo preparations as described above and subsequently fixed and F‐Actin stained with blue phalloidin (pseudocolored gray). Red arrowheads in the right panel point to small, internalized adjuvant particles. Spinning disk confocal images represent a merge of z‐stacks. Images are representative of three independent trials. Fifty cells per trial per condition were analyzed. Scale bars, 5 μm. (c) U‐937 cells were incubated with control or freeze/thaw AlOOH‐lumo preparations and processed as described in (b). The percentage of cells with intracellular AlOOH particles were calculated for three independent trials and the mean represented in the bar graph. *p ≤ .05

AlOOH‐containing vaccine formulations typically consist of antigens adsorbed to a population of particles that is highly heterogeneous in size, typically ranging from 1 to 10 μm in diameter (Hem & Hogenesch, 2007a). To investigate the effect of particle size on the uptake of AlOOH by macrophages, we tested two different preparations of AlOOH with distinct particle size distributions: a preparation displaying a median diameter d(0.5) of 6.6 μm (control), and a preparation of AlOOH treated by repeated freeze‐and‐thaw cycles (freeze/thaw) to favor the formation of large particles, displaying a median diameter d(0.5) of42.2 μm (Figure S1), which has been reported to lead to the loss of vaccine potency (Clapp et al., 2014). Next, AlOOH from control and freeze/thaw preparations were labeled with lumogallion and utilized for internalization assays with U‐937 macrophages. After 4 h of incubation with either preparation of AlOOH‐lumo, macrophages were detached from their wells and labeled with DAPI and anti‐CD11c antibodies for flow cytometry analysis (Figures 5a and S1). As shown in Figure 5b,c, 87 ± 7.5% of the cells incubated with the control AlOOH preparation internalized AlOOH‐lumo particles. However, only 34 ± 15% of macrophages incubated with the freeze /thaw AlOOH preparation contained intracellular AlOOH‐lumo. Most of the internalized AlOOH were smaller particles present in the freeze/thaw preparation, while the large particles remained extracellular, as revealed by the microscopy imaging data from Figure 5b. Altogether, these results confirm that particle size is a critical attribute for adjuvant internalization by APCs.

### 2.3 Internalized AlOOH traffic to functional endolysosomal compartments

**FIGURE 6:**
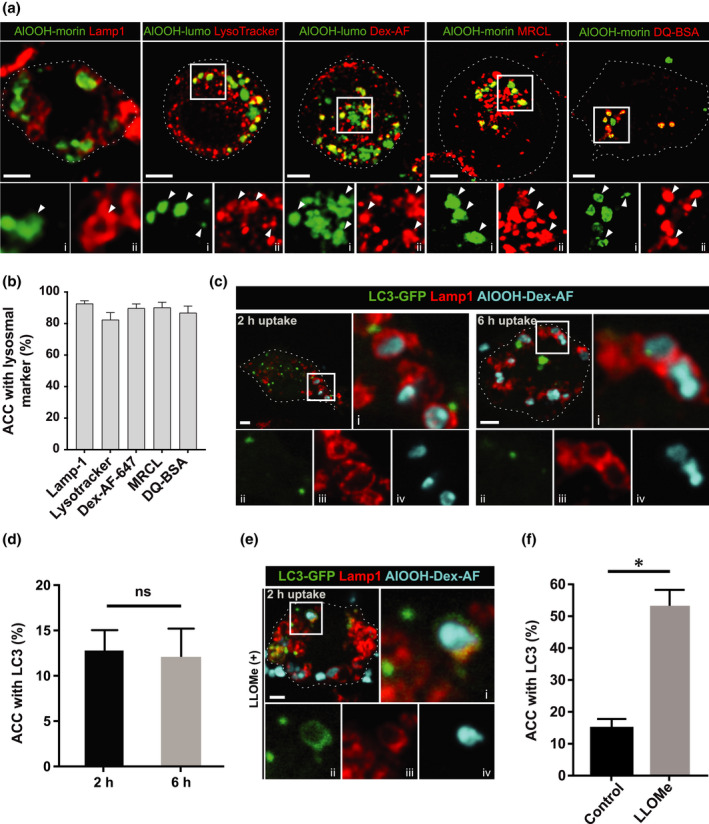
Adjuvant containing compartments acquire endolysosomal properties. (a) U‐937 cells were incubated with AlOOH‐morin or AlOOH‐lumo for 2 h at 37°C to study the association of fluorescent AlOOH particles with the following endolysosomal markers: Lamp‐1 (immunofluorescence), LysoTracker deep red, pre‐loaded 10 kDa Dex‐AF647, magic red cathepsin L (MRCL) or DQ‐red BSA, as described in experimental procedures. Scale bars, 5 μm. (i) and (ii) represent individual fluorescent channels enlarged from the framed area in the merge above. (b) the percentage of ACCs positive for the abovementioned markers are shown in the bar graph and are representative of three independent trials. (c) RAW cells were transiently transfected with an LC3‐GFP construct and 12 h post‐transfection, cells were incubated with AlOOH‐Dex‐AF647 at 37°C for 2 or 6 h and subsequently processed for immunofluorescence against Lamp‐1. (d) the percentage of adjuvant‐containing compartments positive for LC3 at the indicated internalization assay time points, and the mean represented in the bar graph. (e) RAW cells were transiently transfected, incubated with fluorescent adjuvant for 2 h and processed for immunofluorescence as in (c), however, prior to fixation, cells were treated with the lysomotropic agent LLOMe (250 μM) for 30 min. The percentage of ACCs positive for LC3‐GFP are depicted in the bar graph. (f) the percentage of adjuvant containing compartments positive for LC3, and the mean represented in the bar graph. *p ≤ .05. Framed areas are enlarged in (i–iv). Specifically, (i) represents the merge while (ii–iv) represent single fluorescent channels. Spinning disk confocal images correspond to a single z‐plane. Images are representative of three independent trials. Fifty cells per trial per condition were analyzed. Scale bars, 5 μm

We next sought to characterize the intracellular compartment occupied by AlOOH particles in macrophages, which will hereafter be referred to as AlOOH‐containing compartments (ACCs). AlOOH immune adjuvanticity has been associated with the rupture of endosomes, caused by AlOOH‐induced oxidative damage of endosomal membranes, and leading to NLRP3‐inflammasome activation (Eisenbarth et al., 2008; Hogenesch, 2012; Mold et al., 2016; Reinke et al., 2020). Although this may trigger inflammatory responses and the recruitment and activation of APCs at the site of vaccine administration, it may also render endosomal compartments non‐degradative, affecting antigen processing and the presentation capacity of APCs (Trombetta et al., 2003). To shed light on this conundrum, we investigated if ACCs could complete their endosomal maturation and develop endolysosomal degradative properties. As shown in Figure 6a,b, the vast majority of AlOOH‐lumo in U‐973‐cells localized to ACCs positive for endolysosomal markers, as also seen for RAW cells (Figure S2). ACCs were positive for Lysosomal‐associated membrane protein 1 (Lamp‐1) and fused with late endosomes and lysosomes labeled with pre‐loaded Alexa Fluor®‐conjugated dextran, indicating they successfully reach the late endo‐lysosomal maturation stage. This was further confirmed by assessing their capacity to acidify. The acidification of endolysosomes relies on the vacuolar ATPase (v‐ATPase) H^+^ pump, and the low permeability to H^+^ of endolysosomal membranes to sustain a H^+^ gradient. Figure 6a,b show that most of the ACCs analyzed were acidic, since they accumulated the acidotropic fluorescent compound LysoTracker, and hence indicate being delimited by intact membranes able to sustain H^+^ gradients. Moreover, as expected for functional degradative endolysosomal compartments, ACCs tested positive for lysosomal protease activity, as per the chromogenic protease substrates Magic Red and DQ‐red BSA (Figure 6a,b).

Next, we assessed whether ACCs could remain degradative due to the action of autophagy‐mediated repair mechanisms counterbalancing AlOOH’s putative capacity to induce membrane damage (Chauhan et al., 2016; Maejima et al., 2013). To investigate this possibility, we assessed the recruitment of the autophagy protein Microtubule‐associated protein 1A/1B‐light chain 3 (LC3) to ACCs since LC3 binds damaged endocytic compartments, as they are targeted for autophagy‐mediated repair. Figure 6c,d show that LC3‐GFP is seldom recruited to Lamp‐1 positive ACCs, unless the compartments were deliberately damaged by treating macrophages with the lysosomal‐specific membranolytic compound L‐leucyl‐L‐leucine methyl ester (LLOMe) (Figures 6e,f and S3) (Maejima et al., 2013; Thiele & Lipsky, 1990; Uchimoto et al., 1999). To further confirm these results, we resorted to assessing the recruitment of mCherry‐galectin 8 and GFP‐lysenin to ACCs, which also detect damaged compartments (Figure S3a), albeit being sensitive to different levels of membrane damage in endocytic compartments. Galectin‐8 binds to sugar moieties exposed in damaged compartments to target them for selective autophagy. The lysenin probe binds sphingomyelin, a lipid in the luminal leaflet of endolysosomal membranes that readily translocate to the cytosolic face upon small membrane disruptions that are undetectable by the other probes (Thurston et al., 2012). Figure S3b,c shows that neither mCherry‐galectin‐8 nor GFP‐lysenin, associated with ACCs, unless LLOMe was applied to the cells. Altogether our results demonstrate that ACCs are functional degradative endolysosomal compartments.

### 2.4 Unadjuvanted gdPT disrupts and escapes the endolysosomal pathway

**FIGURE 7:**
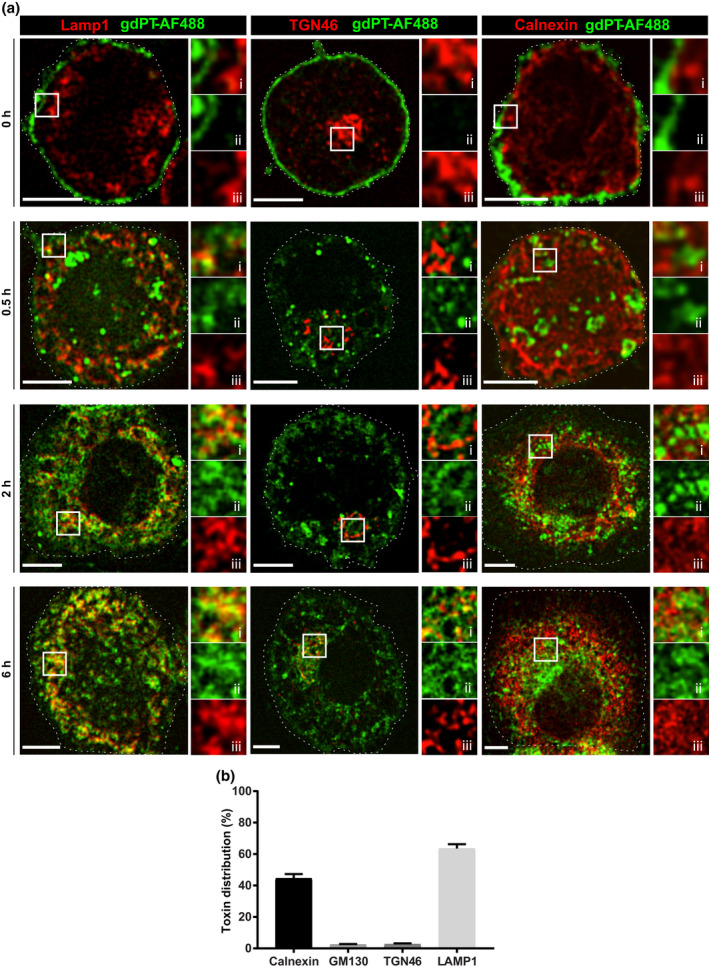
Intracellular trafficking of gdPT. (a) U‐937 cells were allowed to internalize gdPT‐AF488 as described in experimental procedures and fixed at the indicated time points. Cells were immunostained against Lamp‐1, TGN46, and calnexin. Spinning disk confocal images correspond to a single z‐plane. Framed areas are enlarged in (i–iii). Specifically, (i) represents the merge while (ii–iii) represent single fluorescent channels. Images are representative of three independent trials. 50 cells per trial per condition were analyzed. Scale bars, 5 μm. (b) U‐937 cells were incubated with gdPT‐AF488 for 6 h and then processed for immunofluorescence against calnexin, GM130, TGN46, or Lamp‐1. For each free toxin containing compartment, the Manders’ coefficient (M_2_) was determined and if M_2_ was greater than 0.7, and the particle was considered positive for that marker. Data are presented as mean ± SEM of a representative experiment, where at least 10 cells were quantified for each condition

**FIGURE 8:**
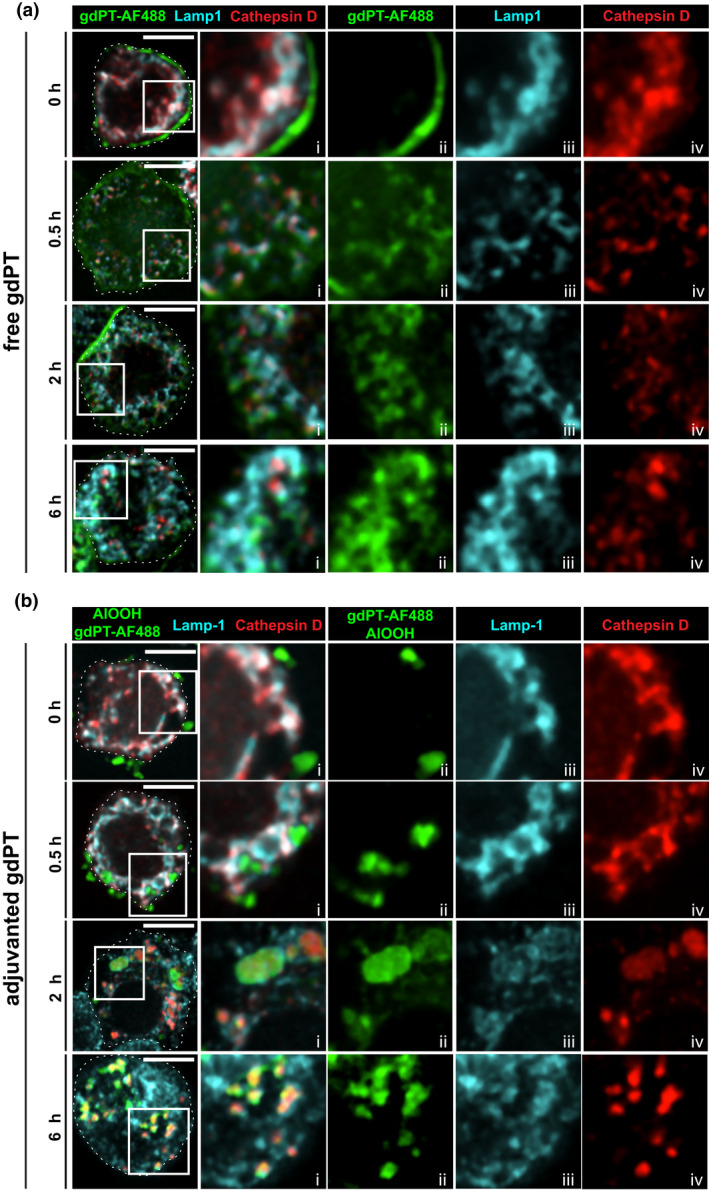
Intracellular trafficking of free and adjuvanted gdPT. U‐937 cells were incubated with either gdPT‐AF488 (a) or AlOOH‐gdPT‐AF488 (b) as described in experimental procedures and fixed at different time points. Cells were subsequently processed for immunostaining against Lamp‐1 and cathepsin D. spinning disk confocal images correspond to a single z‐plane. Framed areas are enlarged in (i–iv). Specifically, (i) represents the merge while (ii–iv) represent single fluorescent channels. Images are representative of three independent trials. Over 100 cells per trial per condition were analyzed. Scale bars, 5 μm

**FIGURE 9:**
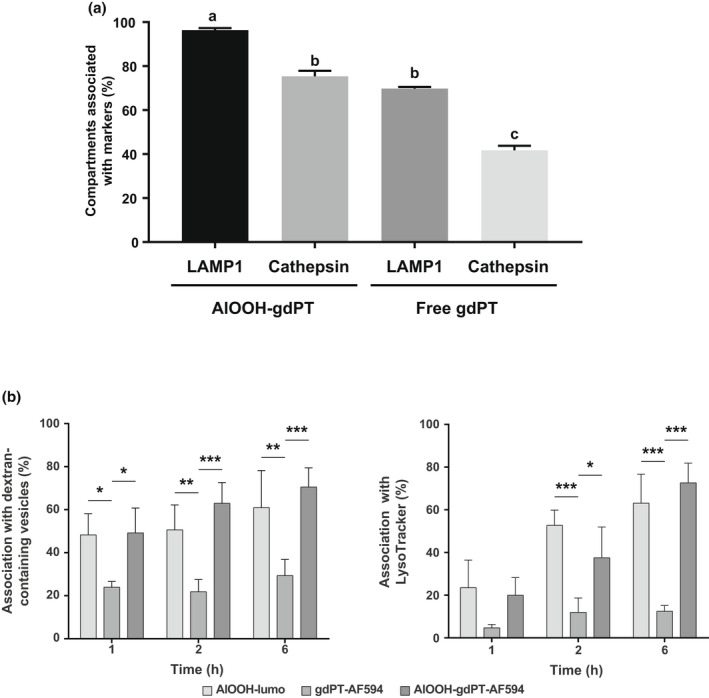
Quantification of the association of free toxin, adjuvanted toxin or adjuvant alone with various intracellular markers. (a) U‐937 cells were incubated with gdPT‐AF488 or AlOOH‐gdPT‐AF488 for 6 h and then immunostained against Lamp‐1 and cathepsin D. For either AlOOH‐gdPT or free gdPT compartment, the Manders’ coefficient (M_2_) was determined and if M_2_ was greater than 0.7, the particle was considered positive for that marker. Data are presented as mean ± SEM of three independent experiments, where 10 cells were quantified for each condition per independent experiment. *p*‐Value ≤0.05. (b) U‐937 macrophages were incubated with AlOOH‐lumo, gdPT‐AF594, or AlOOH‐gdPT‐AF594 for the indicated time points. Percent association of compartments with AlOOH‐lumo, gdPT‐AF594, or AlOOH‐gdPT‐AF594 with dextran (left) or LysoTracker (right) is depicted in the bar graphs. Data are presented as mean ± SEM of three independent experiments. *p ≤ .05, **p ≤ .01, *** *p* ≤ .001

Since ACCs are endowed with endolysosomal degradative properties, it is conceivable that the trafficking of AlOOH‐adsorbed antigenic proteins to ACCs may favor their degradation and processing for antigen presentation, hence contributing to AlOOH adjuvant function. This could be critical for vaccine antigenic components that have the capacity to escape the endo‐phagocytic route, as could be the case of viral particles and bacterial exotoxins (Uribe‐Querol & Rosales, 2017). To investigate this hypothesis, we compared the intracellular fate of free and AlOOH‐adsorbed gdPT in U‐937 and RAW macrophages. This firstly required the characterization of gdPT trafficking, which has not been determined thus far. To follow gdPT in macrophages, we tagged gdPT with Alexa Fluor® dyes (gdPT‐AF; see experimental procedures and Figure S4a,b for details). Briefly, U‐937 macrophages were incubated with gdPT‐AF at 4°C for 30 min to allow for the binding of gdPT‐AF to the cells. The cells were then chased at 37°C, to trigger gdPT‐AF endocytosis, synchronously. As shown in Video S6 and Figure S4b, gdPT‐AF binds neatly to the surface of U‐937 cells (0 h) and is rapidly internalized when conditions are permissive to endocytosis. Next, we ran a similar pulse and chase experiment, but in this case, the cells were fixed at the indicated times and processed for immunofluorescent labeling to assess the trafficking of gdPT‐AF to different organellar compartments. As shown in Figure 7a, after 30 min into the internalization assay, gdPT‐AF moved from the plasma membrane (0 h) into vesicles that partially co‐localize with the late endolysosomal maker Lamp‐1, as expected from a late endosomal stage of endocytic maturation (Figure 7a, left panels). For later time points (2 h and 6 h) gdPT‐AF was found chiefly in endosomal reticular compartments strongly associated with Lamp‐1. These results were further confirmed in RAW cells (Figure S5a). Furthermore, we detected gdPT‐AF associated with the *trans*‐Golgi, but not with the *cis*‐Golgi, cisternae, as per its association with the Golgi markers TGN46 and GM‐130, respectively (Figure 7a, middle and Figure S6a,b). For the later time points examined, gdPT‐AF also localized at the ER, as per its association with the ER membrane chaperone protein calnexin (Figure 7a, right panels and Figure S6b). Thus, the association of gdPT‐AF with endosomal, Golgi, and ER compartments strongly suggest that gdPT follows the retrograde pathway toward the ER, as has previously reported for the wild‐type toxin (Carbonetti, 2010; Kugler et al., 2007; Plaut & Carbonetti, 2008). Nevertheless, most of the gdPT‐AF localized within late endosomal compartments by 6 h after the onset of the internalization assay (Figure 7b), which prompted us to investigate the state of maturation of this gdPT‐AF containing endosomal compartments. As shown in Figures 8 and 9, these Lamp‐1 positive gdPT‐AF compartments were depleted from endolysosomal degradative properties. Indeed, they chiefly exclude the lysosomal protease cathepsin D (Figures 8a and 9a) and did not acidify, as per the lack of accumulation of the acidotropic dye LysoTracker (Figure 9b, right graph). Thus, our results indicate that gdPT hinders the ability of endolysosomal compartments to mature into degradative organelles, a phenomenon that may diminish macrophages’ capacity for processing and presenting gdPT‐derived antigens.

### 2.5 AlOOH divert gdPT to the endo‐lysosomal pathway

**FIGURE 10:**
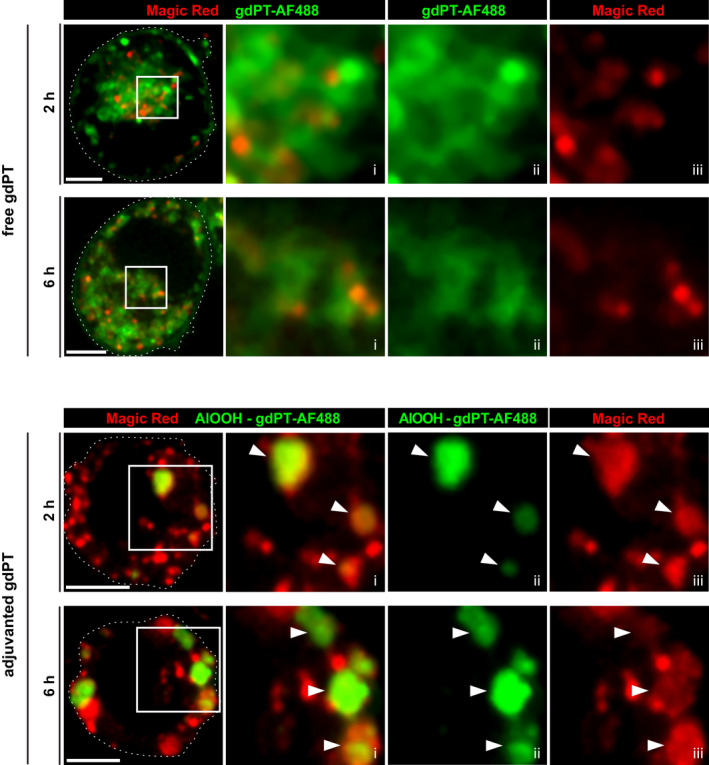
Proteolytic activity in free or adjuvanted gdPT‐containing compartments. U‐937 cells were allowed to internalize either gdPT‐AF488 or AlOOH‐gdPT‐AF488 for the indicated time points and processed for the visualization of cathepsin activity by live cell confocal microscopy using the fluorogenic substrate, magic red, as described in experimental procedures. Representative micrographs of 2 and 6 h of incubation with free or adjuvanted gdPT are shown and are representative of three independent trials. Spinning disk confocal images correspond to a single z‐plane. Framed areas are enlarged in (i–iii). Specifically, (i) represents the merge while (ii–iii) represent single fluorescent channels. Fifty cells per trial per condition were analyzed. White arrowheads point to adjuvanted gdPT positive for the magic red probe. Scale bars, 5 μm

We next investigated the trafficking of gdPT adsorbed to AlOOH (AlOOH‐gdPT‐AF) in U‐937 macrophages. The AlOOH‐gdPT‐AF were produced by incubating gdPT and AlOOH particles for 30 min at room temperature in tris‐buffered saline. The AlOOH‐gdPT‐AF particles recovered were the product of electrostatic interactions, as their formation was reduced in the presence of increasing concentrations of potassium phosphate (Figure S4c,d). Nevertheless, the AlOOH‐gdPT‐AF complex was resistant to multiple washes with PBS buffer and immunostaining conditions and thus, appropriate for our cellular studies. When the AlOOH‐gdPT‐AF particles were internalized by either U‐937 or RAW macrophages, they chiefly localize in Lamp‐1 positive ACCs (Figures 8b and S5b). Unlike for the free gdPT endosomal compartments, ACCs contained cathepsin D (Figure 8b). This was clearly demonstrated by the co‐localization analysis from Figure 9a, showing a significantly higher percentage of cathepsin D co‐localizing with AlOOH‐gdPT‐AF particles than for free gdPT. Moreover, the co‐localization analysis from Figure 9b (left graph) indicated that the compartments containing AlOOH‐gdPT‐AF readily acidify and fuse with late endosomes and lysosomes that were pre‐labeled with fluorescent dextran. Thus, our results reveal a drastic difference between levels of maturation achieved by the adjuvanted and unadjuvanted‐gdPT‐AF containing compartments. Considering this phenomenon, we next sought to investigate if the difference in maturation observed would be reflective of the degradative capacity of either the gdPT or AlOOH‐gdPT containing compartments. As shown by the live cell imaging from Figure 10, while the membrane permeable substrate for cathepsin L, Magic Red, was cleaved and became fluorescent in compartments enclosing AlOOH‐gdPT‐AF, this was not the case for the unadjuvanted gdPT‐AF containing endosomes, therefore indicating that only the former is degradative. Altogether these results demonstrate that unlike free gdPT, the AlOOH‐absorbed gdPT no longer interferes with the maturation and acquisition of degradative properties of its intracellular compartment.

## 3 DISCUSSION

Despite the critical role that aluminum‐based adjuvants play in immune response augmentation, the cellular mechanisms underlying their interactions with APCs, i.e., particle internalization and intracellular trafficking, are poorly understood (Marrack et al., 2009). To investigate the uptake and trafficking of aluminum‐based adjuvants in macrophages, and to determine the impact of adjuvant‐adsorption on the intracellular fate of the pertussis vaccine antigen in vitro, we utilized AlOOH and gdPT as an antigen‐adjuvant model. We show that macrophages can internalize a wide range of AlOOH particle sizes, instead of undergoing abortive phagocytosis as previously reported for antigen‐loaded aluminum adjuvants (Flach et al., 2011), and that most of these internalized particles follow the endo‐lysosomal trafficking, endowing their compartments with a degradative lumen. Importantly, the internalization efficiency of AlOOH in macrophages was drastically reduced when the average size of the particles was increased by freeze and thaw cycles, which induces AlOOH aggregation. This size‐dependent effect may explain the reduction in vaccine immunogenicity observed after subjecting vaccines to freeze–thaw cycles (Clapp et al., 2014), thereby demonstrating that particle size is a critical determinant for AlOOH uptake by macrophages, and confirming previous studies showing the importance of adjuvant particle size in vaccine immunogenicity (Clausi et al., 2008; Shardlow et al., 2017).

We show that the fate of gdPT within macrophages is strictly determined by whether it is adsorbed to AlOOH particles. Hence, several attributes critical to antigen‐adjuvant formulation were identified and a simpler explanation for AlOOH‐based adjuvantation was proposed.

By following the uptake of AlOOH by live‐cell microscopy and utilizing pharmacological inhibitors, we demonstrated that macrophages internalize non‐opsonized AlOOH particles via macropinocytosis. Furthermore, video microscopy revealed that AlOOH particles are internalized at areas of the macrophages surface undergoing copious and continuous membrane ruffling and where the formation of macropinosomes is evident, which is a behavior consistent with the constitutive form of macropinocytosis reported for macrophages and dendritic cells (Doodnauth et al., 2019). Through constitutive macropinocytosis, APCs ceaselessly sample extracellular fluids surveying for antigens (Canton et al., 2016; Sallusto et al., 1995). However, unlike for the macropinocytosis of extracellular fluid, we herein show that AlOOH particles are dragged by filopodia or filopodial‐like extensions toward the ruffling membrane. This particle capturing mechanism has been previously described assisting the opsonic and non‐opsonic phagocytosis of microbes and disparate particles (Flannagan et al., 2010; Jain et al., 2019; Sallusto et al., 1995), and has been shown to be dependent on filopodial pulling and contraction mechanisms controlled by various myosin actin motors and actin tread‐milling (Alieva et al., 2019; Horsthemke et al., 2017; Kress et al., 2007). Multiple sensing and binding receptors mediate filopodia environmental scouting and particle‐capturing functions, including integrins, cadherins, and phagocytic receptors (Alieva et al., 2019; Chang et al., 2016; Horsthemke et al., 2017). In this regard, it has been reported that AlOOH‐polymer nanoparticles can bind the scavenger receptor A (Jiang et al., 2018). Nevertheless, in our hands, the blocking of integrins, scavenger receptors, or complement receptor 3 (CD11b/CD18) impacted neither the binding (data not shown) nor the internalization of AlOOH particles by RAW and U‐937 macrophages. Consequently, we hypothesize that the binding of AlOOH particles to filopodia may lean on the highly adsorptive surface of AlOOH, which may allow for multiple and diverse binding interactions with molecular moieties expressed at the filopodial surface. We speculate that such putative binding properties could mediate the nonselective opsonization of AlOOH and the engagement of opsonic phagocytosis, in a physiological context. Accordingly, we showed that the incubation of AlOOH with nonspecific IgG significantly enhanced the capture and uptake of adjuvant particles by RAW and U‐937 macrophages. To the best of our knowledge, this is the first time that the adsorption of a nonspecific opsonin is shown to enhance adjuvant phagocytosis, suggesting a key role of immunoglobulin‐induced uptake of AlOOH particles in vivo. While antigen‐specific opsonization is well documented in the literature, the ability of AlOOH to engage nonspecific adsorption of opsonins in vivo warrants further investigation.

We show that AlOOH particles traffic to intracellular compartments that we termed ACCs to distinguish them from macropinosomes, which designates compartments carrying bulk liquid, and from phagosomes, which are formed upon a phagocytic event. We demonstrated that ACCs mature acquiring endolysosomal features and degradative properties. Indeed, unlike for the extensively reported lysosomalytic effect of AlOOH intracellular compartments in APCs (Danielsson & Eriksson, 2021), our results show that only a small proportion of ACCs are damaged compartments, targeted by autophagy and not acidic. As a matter of fact, the AlOOH lysosomoytic effect that has been associated with the activation of the NLRP3 inflammasome, and considered key for AlOOH adjuvancy, was shown exclusively in the case of APCs primed with LPS (Hornung et al., 2008; Li et al., 2007; Lima et al., 2013), a condition not explored in this study, since adjuvanted subunit vaccine formulations normally contain no LPS or very low levels of residual endotoxins (Brito & Singh, 2011).

Assessing the impact of AlOOH‐adsorption on gdPT intracellular trafficking required first to characterize the trafficking of the unadjuvanted gdPT in macrophages. We found that unadjuvanted gdPT, despite having its ribosylation‐based toxicity disarmed, is still capable of disrupting endosomal maturation and subverting the trafficking retrogradely to the Golgi cisternae and then to the endoplasmic reticulum, likely following the pathway reported for the wild‐type PTx (el Baya et al., 1997; Plaut & Carbonetti, 2008). To the best of our knowledge, our study is the first one describing the trafficking of gdPT and reporting its capacity of disrupting endosomal maturation. On the other hand, the adsorption of gdPT to AlOOH hampers its ability to disrupt and escape the endocytic pathway, and this could be the consequence of AlOOH keeping gdPT from interacting with the ACCs membrane. Consequently, different from unadjuvanted gdPT containing compartments, AlOOH‐gdPT containing compartments are degradative organelles, and hence, possibly capable of antigen processing and MHC‐II presentation (Ghimire et al., 2012). This indicates that by simply adsorbing antigens that bear the capacity to escape and/or disrupt the endocytic pathway to AlOOH, these antigens can be re‐routed to degradative compartments and eventually presented to T cells as MHC‐II‐peptide complexes. Importantly, these putative mechanisms could also apply to other vaccine antigens with the potential of disrupting intracellular trafficking. Nevertheless, more studies are required to understand the mechanism behind this phenomenon and how it contributes to aluminum adjuvancy.

In our study, gdPT was readily adsorbed to AlOOH particles. However, it has been reported that immunopotentiation does not always correlate with a high degree of adsorption and could also be induced when antigens are not adsorbed to the adjuvant. Romero Mendez et al. observed that non‐adsorbed antigens were entrapped in void spaces within the adjuvant aggregates and that this was enough to induce uptake of antigens by APCs (Romero Mendez et al., 2007). Whether entrapped non‐adsorbed antigens follow the same intracellular fate within APCs to that of adsorbed antigens remains unknown but warrants further investigation. To answer this important question future studies should look at the uptake and phagocytosis of gdPT formulated with AlPO_4_ adjuvant where antigen adsorption is expected to be diminished by electrostatic repulsion between the negatively charged gdPT and AlPO_4_ at neutral pH.

Our results show that there are several attributes likely to be critical for an antigen‐AlOOH formulation that may need to be monitored and controlled throughout vaccine development. These include: (i) the size of the AlOOH particle which may affect whether a particle can be taken up at all; (ii) the capacity to adsorb IgG or other opsonins nonspecifically; and (iii) the stability of adjuvant‐antigen absorption during formulation and after vaccine administration. Adsorption of antigen can be specifically controlled during the formulation process and could have a profound impact on the type of immune response elicited, especially for antigens like gdPT. Control of such attributes will provide better predictability of the vaccine response in terms of safety and immunogenicity. While the mechanistic role of aluminum‐based adjuvants has been the subject of much discussion (Oleszycka & Lavelle, 2014), we speculate that the simple process of particle size range optimization and the ability to direct immunity by uptake and trafficking toward degradative endocytic compartments might be a central mechanism underpinning the AlOOH immunopotentiation effect at the cellular level. Indeed, others have demonstrated the capacity of AlOOH to induce immunity independently of NLRP3/caspase1 (Franchi & Nunez, 2008; Marrack et al., 2009; McKee et al., 2009; Oleszycka & Lavelle, 2014). Nevertheless, our findings do not rule out a contribution of NLRP3 inflammasome to AlOOH adjuvancy, since we detected AlOOH lysosomalytic activity in unprimed macrophages affecting a reduced number of ACC, which could yet activate NLRP3, allowing for its immunomodulatory contribution to adjuvancy.

## 4 EXPERIMENTAL PROCEDURES

### 4.1 Cell lines and culture conditions

RAW 264.7 murine macrophages (ATCC TIB‐71™) and RAW cells stably expressing LifeAct‐RFP (Dr. Rene Harrison, University of Toronto) were cultured in DMEM medium (Wisent Inc., Canada) supplemented with 10% heat‐inactivated fetal bovine serum (FBS) (Wisent Inc.). U‐937 human macrophage‐like cells (ATCC CRL‐1593.2™) were cultured routinely in suspension in RPMI 1640 medium (Gibco, Thermo Fisher Scientific, Canada) supplemented with 10% FBS (Gibco, Thermo Fisher Scientific). To induce differentiation and adherence, cells were incubated with 100 ng ml^−1^ phorbol‐12‐myristate‐13‐acetate (PMA, cat# P1585, Sigma Aldrich) for 48 h. For brightfield, confocal, and scanning electron microscopy, U‐937 cells were directly grown and differentiated as above onto glass coverslips in multi‐well plates. All cell lines were maintained at 37°C in a CO_2_ incubator.

### 4.2 Plasmids and transfection

The construct GFP‐LyseninW20A was kindly provided by Dr. Jonathan Canton, University of Calgary (Calgary Alberta). LC3‐GFP was kindly provided by Dr. Maria Isabel Colombo (IHEM‐UNCuyo, Argentina) and galectin‐8‐mCherry was a gift from Dr. Felix Randow (Medical Research Council, Cambridge, UK).

For transfections, RAW cells were seeded on glass coverslips to 60–70% confluency and transfected using Lipofectamine™ LTX reagent with PLUS™ reagent (cat# 15338100, Thermo Fisher Scientific) according to the manufacturer’s instructions. At 14 h post‐transfection, cells were employed to assess the membrane integrity of ACCs.

### 4.3 Antibodies

The following antibodies were used for immunofluorescence or flow cytometry assays: mouse anti‐human CD11c monoclonal antibody (cat# 14‐0116‐82, Thermo Fisher Scientific); rat anti‐mouse Lamp‐1 antibody (clone 1D4B, Developmental Studies Hybridoma Bank); mouse anti‐human Lamp‐1 antibody (clone H4A3, Developmental Studies Hybridoma Bank); mouse anti‐human GM130 antibody (cat# 610822, BD Biosciences); rabbit anti‐human calnexin antibody (cat# ab112995, abcam); rabbit anti‐bordetella pertussis toxin antibody (cat# ab188414, abcam); rabbit anti‐human TGN46 antibody (cat# ab50595, abcam); rabbit anti‐cathepsin D antibody (cat# 219361, Millipore Sigma); Cy3‐donkey anti‐rat IgG (cat# 712–165‐153, Jackson Immunoresearch); donkey anti‐rabbit Alexa Fluor 647 (cat# A31573, Invitrogen); donkey anti‐rabbit IgG Alexa Fluor 555 (cat# A31572, Life Technologies); goat anti‐mouse Alexa Fluor 488 (cat# 11029, Thermo Fisher Scientific); goat anti‐rabbit Alexa Fluor 647 (cat# A21245, Thermo Fisher Scientific); goat anti‐mouse Alexa Fluor 647 (cat# A21235, Thermo Fisher Scientific); donkey anti‐mouse Alexa Fluor 555 (cat# A31570, Thermo Fisher Scientific). The following antibodies were used during blocking and opsonization assays: rat anti‐mouse CD11b (blocking antibody, clone: M1/70, cat# AB‐467108, Thermo Fisher Scientific) and human IgG (opsonization, I8640 or I4506, Millipore Sigma).

### 4.4 Fluorescent labeling of AlOOH particles and gdPT

AlOOH‐lumo was prepared as described elsewhere (Mile et al., 2015). Briefly, 100 μl of [4‐chloro‐3‐(2,4‐dihydroxyphenylazo)‐2‐hydroxybenzene‐1‐sulphonic acid] stock solution (0.5 mM diluted in water; Lumogallion, cat# 215156480, MP Biomedicals, Solon, OH, USA) was co‐incubated with 5 mg of AlOOH (ALHYDROGEL® “85” 2%, Brenntag Biosector A/S, Frederikssund, Denmark) in 1 ml of RPMI media in a rotating platform for 24 h at RT. AlOOH‐morin was prepared by incubating 100 ul of [2′,3,4′,5,7‐penta hydroxyflavone] (morin) stock solution (250 μM diluted in water; cat # 69870, Millipore Sigma) with 0.5 mg AlOOH in 1 ml of 10 mM Tris–HCl pH 7.4 in a rotating platform for 24 h at RT. For AlOOH‐Dex‐AF, 5 mg of AlOOH was incubated with either 100 ul Dextran, Alexa Fluor™ 488 (Dextran, Alexa Fluor 488 10,000 MW, anionic, fixable, cat# D22910, Thermo Fisher Scientific) or Dextran, Alexa Fluor™ 647 (Dextran, Alexa Fluor 647 10,000 MW, anionic, fixable, cat# D22914, Thermo Fisher Scientific), both at a stock concentration of 0.5 mg ml^−1^ in 1 ml tris‐buffered saline (TBS) 1X for 24 h in a rotating platform at RT. After labeling, each preparation was washed three times with phosphate‐buffered saline (PBS) 1X at 12,000*g* for 3 min each and stored for several months at 4°C without losing fluorescence.

Purified genetically detoxified pertussis toxin (gdPT) was produced by Sanofi Pasteur Ltd. Canada. gdPT was labeled with Alexa Fluor dyes (gdPT‐AF). Briefly, for Alexa Fluor 488–labeled gdPT, 470 μg of gdPT was incubated with 3 μl of Alexa Fluor reagent (Alexa Fluor™ 488 Carboxylic acid Succinimidyl ester, 25 μg ml^−1^, cat# A20100, Invitrogen) in 0.1 M sodium bicarbonate, pH 8.3, and placed at RT for 12 h on a rotating platform. Unconjugated dye was quenched with 0.15 M glycine, pH 8.5, and labeled gdPT dialyzed against PBS (1X) using a 10 kDa MWCO dialysis cassette (Slide‐A‐Lyzer™ Dialysis Cassettes, cat# 66380, Thermo Fisher Scientific), overnight at RT. gdPT‐AF was aliquoted and stored at −80°C until use. Alternatively, gdPT was labeled with the Alexa Fluor™ 594 protein labeling kit (cat# A10239, Invitrogen) following the manufacturer’s instructions.

### 4.5 Effect of phosphate buffer on the adsorption of gdPT to AlOOH particles

To investigate the effect of electrostatic interactions, the adsorption of gdPT to AlOOH was measured in the presence of increasing concentration of sodium phosphate buffer pH 7.4. Samples of gdPT and AlOOH were mixed on an orbital mixer for 30 min at RT. The samples were then centrifuged for 5 min at 4000*g*. The supernatants containing the non‐adsorbed protein were collected and the gdPT concentration was measured by UV absorption spectroscopy at 280 nm in an Agilent 8453 UV/VIS spectrophotometer.

### 4.6 Adsorption of fluorescent gdPT to AlOOH particles

gdPT‐AF was adsorbed to AlOOH particles (AlOOH‐gdPT‐AF) by co‐incubating 0.6 mg ml^−1^ AlOOH with 40 μg ml^−1^ gdPT‐AF in 1 ml TBS (1X) at RT for 30 min on a rotating platform. AlOOH‐gdPT‐AF were washed with 1 ml TBS (1X) three times at 12,000*g* for 3 min each, to remove the excess of unbound gdPT, and subsequently stored at 4°C for no more than 1 week. Following internalization assays, immunostaining, and mounting of coverslips, slides were visualized within 1 week otherwise fluorescent toxin leach out from their original compartment.

### 4.7 Freeze and thaw treatment of adjuvant particles

To analyze the impact of particle size on AlOOH internalization by macrophages, AlOOH particles were subjected to five consecutive cycles of freezing/thawing (−80°C for 15 min/37°C for 5 min) and when required, this was followed by lumogallion labeling as described above. The particle size distribution was measured by laser diffraction in a Mastersizer 2000 equipped with a Hydro 2000S sample dispersion unit (Malvern Instruments Ltd.). The results were processed by volume and the data compared by the median diameter d(0.5) which correspond to the diameter below which 50% of the particles are distributed by volume.

### 4.8 Internalization assays of AlOOH, gdPT, and AlOOH‐gdPT

2–10 μg ml^−1^ AlOOH or AlOOH‐gdPT were centrifuged on RAW or U‐937 cells at 300*g* for 5 min at 10°C. Subsequently, cells were washed with ice‐cold Dulbecco’s phosphate‐buffered saline (D‐PBS) 1X, three times, and incubated at 37°C for various time points with either DMEM or RPMI +10% FBS.

20–40 μg ml^−1^ gdPT (unlabeled or fluorescently labeled) were added to RAW or U‐937 cells (previously cooled at 4°C for 10 min) and subsequently incubated at 4°C for 30 min to prevent internalization. Cells were washed with ice‐cold Dulbecco’s phosphate‐buffered saline (D‐PBS) 1X, three times, and incubated for various time points at 37°C with either DMEM or RPMI +10% FBS.

### 4.9 Internal/external labeling of AlOOH particles

Following internalization assays, and prior to fixation with 4% paraformaldehyde (PFA), cells were cooled down at 4°C for 10 min. Afterwards, ice‐cold Dextran‐AF647 (0.01 mg ml^−1^), diluted in PBS (1X), was added to cells for 10 min at 4°C, rocking plates gently by hand every 2 min. Then, cells were washed three times with ice‐cold PBS (1X) and processed employing the different methodologies described in the sections below.

### 4.10 Immunofluorescence and fluorescence labeling of endocytic compartments

For immunolabeling of endogenous proteins, RAW or U‐937 cells were grown on glass coverslips and after internalization assays, fixed in 4% PFA for 15 min at RT. For Lamp‐1 immunostaining, cells were permeabilized with methanol at −20°C for 10 min, blocked for 30 min at RT with 2.5% bovine serum albumin (BSA) diluted in PBS (1X). Afterwards, cells were incubated with a 1/100 dilution of anti‐Lamp‐1 antibodies for 1 h at RT, washed three times with PBS (1X), incubated for 1.5 h at RT with 1/1500 fluorescently tagged secondary antibodies, and subjected to another round of PBS (1X) washes. For all other immunostainings, cells were permeabilized with 0.1% Triton X‐100 in PBS (1X) for 10 min at RT, blocked with 2.5% BSA for 30 min at RT, and afterwards, incubated with a 1/500 dilution of anti‐PT antibody for 1 h at RT; 1/300 dilution of anti‐GM130 antibody overnight at 4°C; a 1/500 dilution of anti‐calnexin antibody for 1.5 h at RT; a 1/300 dilution of anti‐TGN46 antibody overnight at 4°C; or a 1/1000 dilution anti‐cathepsin D antibody for 1 h at RT. After three washes with PBS (1X), cells were incubated with the corresponding secondary antibodies for 1.5 h and washed again. Cells were mounted with Dako fluorescent mounting medium (cat# S3023, Aligent, Canada).

Actin labeling was performed by two different methodologies. For fixed cell imaging, RAW or U‐937 cells were grown in glass coverslips and after internalization assays, fixed in 4% PFA and permeabilized with 0.1% Triton X‐100 in PBS (1X) for 10 min at RT. Afterwards, cells were washed three times with PBS (1X) and then incubated with a 1/500 dilution of either Alexa Fluor™ 647 Phalloidin (cat# A22287, Thermo Fisher Scientific) or Alexa Fluor™ 405 Phalloidin (cat# A30104, Thermo Fisher Scientific) for 30 min at 37°C. Finally, cells were washed three times with D‐PBS (1X) and mounted with Dako fluorescent mounting medium. For live cell imaging, RAW or U‐937 cells were seeded on Nunc Lab‐Tek Chambered Coverglass (Thermo Fisher Scientific) and incubated with 500 nM of SiR actin (cat#: CY‐SC001, Cytoskeleton Inc.) for 4 h. Then, cells were washed three times with PBS (1X), incubated with either DMEM or RPMI +10% FBS prior to internalization assays. Cells were visualized in live cell imaging solution (Thermo Fisher Scientific) using Chamlide magnetic chambers (Quorum Technologies Inc., Guelph, ON, Canada).

Dextran pre‐loading was performed by incubating the cells with 0.1 mg ml^−1^ Dextran, Alexa Fluor™ 647 diluted in DMEM or RPMI supplemented with 10% FBS for 1 h at 37°C. Afterwards, cells were washed three times with PBS (1X) and then re‐incubated with DMEM or RPMI supplemented with 10% FBS for 2 h at 37°C prior to internalization assays with AlOOH preparations.

### 4.11 Labeling of acidic compartments and assessment of degradative capacity

For labeling of acidic compartments, 1 h before the end of internalization assays, RAW or U‐937 cells were incubated with 1 μM LysoTracker™ Deep Red (cat# L12492, Thermo Fisher Scientific). Cells were washed, fixed, and mounted as described before.

For labeling of degradative compartments, two methodologies were employed. First, U‐937 cells lysosomes were pre‐loaded with 10 μg ml^−1^ DQ™ Red BSA (cat# D12051, Invitrogen) for 12 h at 37°C. Cells were washed twice with D‐PBS to remove media containing DQ‐red BSA and incubated with complete pre‐warmed culture media for 30 min. Subsequently, cells were incubated with AlOOH as described above in the subsection “internalization assays” and at the end of the assay, cells were washed, fixed, and mounted. Second, RAW or U‐937 cells were incubated with AlOOH, AlOOH‐gdPT‐AF488, or gdPT‐AF488, and 15 min before the end of internalization assay, the cells were labeled with Magic Red Cathepsin L Assay Kit (cat# 941, Immunochemistry Technologies LLC, USA), as per manufacturer’s instructions. Cells were washed once with warm PBS (1X) and visualized in live cell imaging solution.

### 4.12 Inhibitor treatments

To study the internalization mechanism of AlOOH by macrophages, several inhibitors of micropinocytosis and phagocytosis were employed at the following concentrations: 100 μM EIPA ([5‐(N‐ethyl‐N‐isopropyl)] amiloride, cat# A3085, Millipore Sigma), 50 μM LY294002 (cat # 19–142, Millipore Sigma), 0.25 μM Latrunculin A (cat# L5163, Millipore Sigma), 0.01 mg ml^−1^ CD11b blocking antibody, 100 μg ml^−1^ fucoidan (cat# F8190, Millipore Sigma), and 0.1 mg ml^−1^ RGD peptides (Arg‐Gly‐Asp, cat# A8052, Millipore Sigma). Methanol, DMSO, IgG2 isotype control antibody, DMEM, or RPMI were used as control vehicles, respectively. Briefly, RAW or U‐937 cells on glass coverslips were pre‐incubated with the different inhibitors or controls diluted in DMEM or RPMI for 1 h at 37°C, except for Latrunculin A which was incubated for 15 min prior to internalization assays. After 2 h of internalization, AlOOH‐lumo particles were differentially labeled to distinguish between external and internal adjuvant particles as described above in the subsection “Internal/external labeling of AlOOH particles.” Afterwards, cells were fixed with 4% PFA and immunostained against Lamp‐1 as described above.

### 4.13 Sterile damage assay

To induce lysosomal rupture in naïve RAW cells, the cells were incubated with 250 μM L‐leucyl‐L‐leucine methyl ester (LLOMe, cat# 4000725.0005, Bachem, USA) at 37°C for 30 min. To induce lysosomal rupture in cells that have internalized fluorescent AlOOH particles, cells were treated with LLOMe as above, 30 min before the end of the internalization assay. Afterwards, cells were washed three times with PBS (1X), fixed with 4% PFA and then immunostained against Lamp‐1 as described above.

### 4.14 Scanning electron microscopy

Following internalization assays, RAW or U‐937 cells were fixed in 2% glutaraldehyde in 0.1 M sodium cacodylate buffer (pH 7.4) for 2 h at RT. Cells were then washed three times for 5 min with 0.1 M cacodylate buffer (pH 7.4), followed by a post‐fixation with 1% OsO_4_ for 1 h at RT. Cells were washed three times again as described above and incubated for 30 min in 1% tannic acid at RT, followed by another 30 min incubation with 1% OsO_4_ and three more washes. Cells were then stained with 4% uranyl acetate for 30 min in the dark and washed with double‐distilled water for 10 min, three times. Cells were dehydrated in ethanol and sputter‐coated with heavy metals. Images were acquired using a scanning electron microscope (Hitachi S530) and captured and processed using the Quartz PCI software (Quartz Imaging Corporation, Vancouver, BC, Canada).

### 4.15 Confocal and brightfield live‐cell imaging and analysis

Confocal live‐cell imaging was acquired with a Quorum WaveFX spinning disk confocal microscope (details listed below). Internalization assays were performed using a stage incubator (Live Cell Instrument) set at 37°C with 5% CO_2_. Brightfield time‐lapse imaging were acquired in an Etaluma LS720 Live Cell Microscope (Etaluma San Diego, USA) inside of a CO_2_ incubator. Cells were incubated in Live Cell Imaging Solution (1X) (HEPES buffered physiological saline, cat# A14291DJ, Thermo Fisher Scientific) for the duration of the movies. Movies were processed with Adobe Photoshop and Illustrator (Adobe Systems Inc.).

### 4.16 Spinning disk confocal fluorescent microscopy and image analysis

Images were acquired with a Leica DMI6000B spinning‐disk confocal microscope by Quorum Technologies, Inc. (Guelph, Ontario, Canada), equipped with an ORCA‐R^2^ camera or an EM‐CCD Hamamatsu camera (Hamamatsu Photonics, Japan), and a 63 × 1.4 NA oil immersion objective. The acquisition was controlled by Metamorph software (Molecular Devices, LLC). Image processing, deconvolution (utilizing calculated point spread functions, 90% confidence interval, 20 iterations), and analysis were performed using Volocity 6.1.2 software (Quorum Technologies Inc., Guelph, ON, Canada) and ImageJ (U.S. National Institutes of Health, Bethesda, Maryland, USA). Images were processed with Adobe Photoshop and Illustrator (Adobe Systems Inc.).

### 4.17 Quantifications of microscopy images with Volocity®

To determine the percentage of internal adjuvant particles positive for Lamp‐1 or LC3 within RAW and U‐937 cells, Manders’ co‐occurrence analysis was performed in Volocity® software by adapting a protocol from Lancaster et al. (2021). Plasma membrane‐associated adjuvant particles were eliminated from the quantification through the removal of z‐stacks at the top of the cell and careful drawing of the region of interest around the cell. Touching internal particles were separated by Volocity using an object size guide of 6.11 μm^3^ for RAW cells, and 0.4 μm^3^ for U‐937 cells, as determined by measuring the average volume of individual particles from at least 18 images. Adjuvant particles were considered to co‐occur with the markers when M_2_ (channel 2 corresponding to adjuvant fluorescence) was determined was greater than 0.7. The average percentage of ACCs positive for Lamp‐1 or LC3 was reported. Data corresponds to three independent experiments, where 13–30 cells were quantified for the indicated conditions per independent experiment.

To determine the percentage of adjuvant‐gdPT‐AF488 and gdPT‐AF488 positive for Lamp‐1, cathepsin, calnexin, GM130, or TGN46, Manders’ co‐occurrence analysis was performed as indicated above. Images were deconvolved (90% confidence limit) prior to analysis. Touching AlOOH‐gdPT or free gdPT were separated using an object size guide of 0.29 μm^3^. Particles were considered to co‐occur with the markers when the M_2_ (channel 2 corresponding to gdPT fluorescence) was determined to be greater than 0.7. The average percentage of AlOOH‐gdPT and gdPT containing compartments positive for Lamp‐1, cathepsin, calnexin, GM130, or TGN46 was reported and based on 10 cells for each marker per independent experiment.

To determine the effect of the different inhibitor treatments on the internalization of AlOOH‐lumo particles by macrophages, the adjuvant particle uptake index was calculated by normalizing the average volume of adjuvant internalized in treated cells by the corresponding value from untreated cells. Briefly, Volocity^Ⓡ^ was set to identify AlOOH‐lumo but negative for Dex‐AF647 fluorescence (i.e., internalized particles), utilizing an object size guide of 0.20 μm^3^, estimated by determining the average volume of adjuvant particles for at least 50 images. A Volocity^Ⓡ^ algorithm calculated the total volume of intracellular particles in each cell by determining the number of voxels within each particle and the voxel’s volume of the microscope system.

### 4.18 Flow cytometry

Following AlOOH internalizations assays, macrophages were detached washed, and fixed with 4% PFA at 4°C, for 1 h. Afterwards, cells were blocked in 5% skim milk in PBS (1X), and incubated with a 1/100 dilution of anti‐CD11c primary antibody overnight at 4°C on a rotating platform. Subsequently, cells were washed three times with PBS and incubated with fluorescently tagged secondary antibody diluted 1/1000 for 1 h, and with a 1/500 dilution of [4′,6‐Diamidino‐2‐Phenylindole, Dihydrochloride] (DAPI, cat# D1306, Thermo Fisher Scientific, Invitrogen) for 15 min. Flow cytometry analyses were performed in a BD LSRFortessa cell analyzer (BD Biosciences) controlled by BD FACSDIVA (BD Biosciences). Data was analyzed using FlowJo 7.0.2 (BD Biosciences).

### 4.19 SDS‐PAGE and in‐gel fluorescence

3–5 μg of gdPT‐AF488 were mixed with Laemmli’s sample buffer (cat# 1610747, Biorad, Canada) and 2 mM dithiothreitol (DTT, cat# DTT‐RO, Millipore Sigma), and heated at 100°C for 5 min prior to electrophoresis in 16% tris‐glycine polyacrylamide gels for 1.5 h at 120 V. Gels were washed once with milliQ water for 10 min prior to in‐gel fluorescence using the PharosFX imaging system (Biorad). Data was analyzed using the Quantity One software. Afterwards, protein bands were stained in the same gels with InstantBlue™ (cat# ab119211, Abcam) overnight at RT. After 5 washes with milliQ water for 10 min each, protein bands were visualized using the GelDoc imaging system (Biorad). Data was analyzed using the Image Lab software.

### 4.20 Statistical analysis

Unless otherwise stated, data are presented as mean ± SEM of three independent experiments. Statistical analysis was carried out using the Prism 9.0.2 software (GraphPad, La Jolla, CA). Data were assumed to be normally distributed. Two conditions were statistically compared using an unpaired, two‐tailed Student’s *t* test or a nested *t* test, while multiple conditions were compared utilizing a one‐way ANOVA with Tukey’s post hoc test, a one‐way ANOVA with Dunnett’s post hoc test or a nested one‐way ANOVA with Dunnett’s post hoc test. For all the statistical tests performed, *p*‐values ≤ .05 were considered statistically significant.

## CONFLICT OF INTEREST

SFA and RHB are employees of Sanofi Pasteur Ltd. Canada and may hold shares and/or stock options. JRJ‐F, MCG, CYH, SM, CEL, RJB, and MRT declare no competing interests.

## AUTHOR CONTRIBUTIONS

Javier R. Jaldin‐Fincati, Roberto J. Bothelo, Salvador F. Ausar, Roger H. Brooks, and Mauricio R. Terebiznik are responsible for overall design of this study. Javier R. Jaldin‐Fincati, Serene Moussaoui, Maria Cecilia Gimenez, and Cheuk Y Ho carried out the experiments, data analysis, and interpretation. Charlene E Lancaster participated in data analysis. Javier R. Jaldin‐Fincati, Roberto J. Bothelo, Roger H. Brooks, Salvador F. Ausar, Serene Moussaoui, Maria Cecilia Gimenez, and Mauricio R. Terebiznik wrote the paper. All authors gave approval to the final version of the paper.

## ETHICS STATEMENT

The studies described obtained are exempted from ethics approval by Institutional Review Board as no animals or human samples were used, except cell lines.

## Supporting information


Figures S1‐S6

Video S1

Video S2

Video S3

Video S4

Video S5

Video S6

## Data Availability

Data available on request from the authors.
