# Supplementary material for: Aluminum hydroxide adjuvant diverts the uptake and trafficking of genetically detoxified pertussis toxin to lysosomes in macrophages
Source: Mol Microbiol. 2022 Apr 7;117(5):1173–95. doi: 10.1111/mmi.14900 (PMC9321756; doi:10.1111/mmi.14900)
Supplement: Supplementary file 1 — Figures S1‐S6 [file MMI-117-1173-s003.pdf]

Supplementary Figure 1

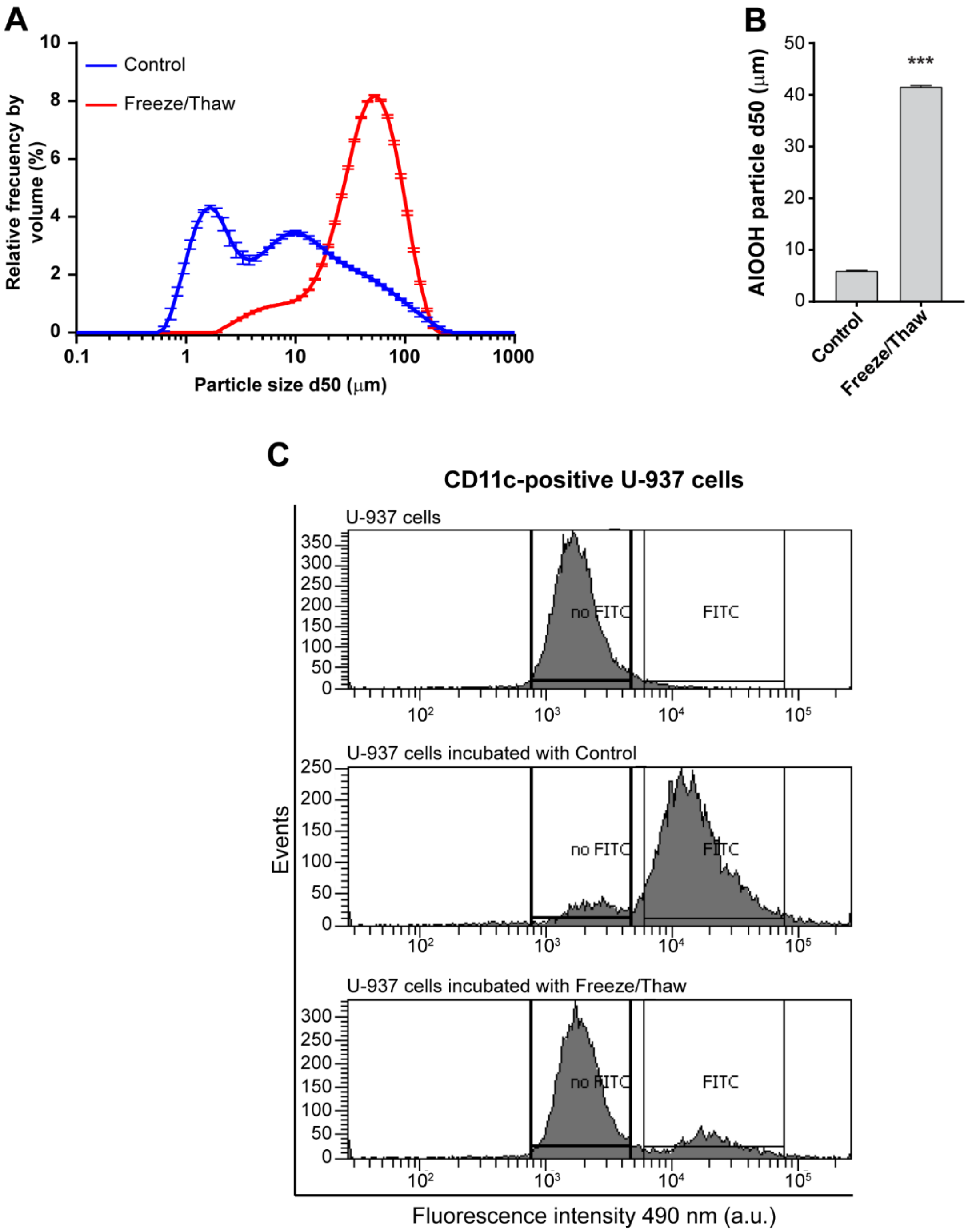

Supplementary Figure 1. The effect of freeze/thaw events on particle size distribution and internalization. A) Plot depicting the difference in size distribution between untreated AlOOH (control) and AlOOH after multiple freeze/thaw cycles (freeze/thaw). Particle size was measured

by laser diffraction. B) Bar graph showing median particle diameter (d50) for both control and freeze/thaw adjuvant preparations. ***p ≤ 0.001. C) U-937 cells were incubated with AlOOH-lumo (control) or AlOOH-lumo after 5 freeze/thaw cycles (freeze/thaw) and processed for flow cytometry. The histograms present CD11c-positive U-937 cells (events) as a function of the fluorescence intensity of the lumogallion emission wavelength (490nm).

Supplementary Figure 2

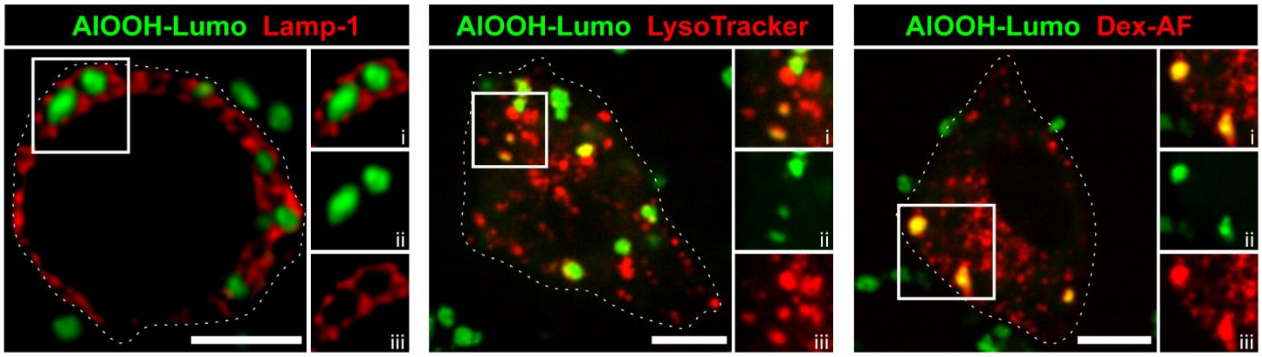

Supplementary Figure 2. ACCs in RAW cells acquire endolysosomal properties. RAW cells were incubated with AlOOH-lumo for 2 h at 37 °C to study the association of fluorescent AlOOH particles with the endolysosomal markers Lamp-1 (immunofluorescence), LysoTracker Deep Red, and pre-loaded 10 kDa Dex-AF647, as described in experimental procedures. Framed areas are enlarged to the right (i-iii). (i) represents the merge and (ii-iii) represent individual channels. Spinning disk confocal images correspond to a single z-plane. Images are representative of three independent trials. 50 cells per trial per condition were analyzed. Scale bars, 5µm.

Supplementary Figure 3

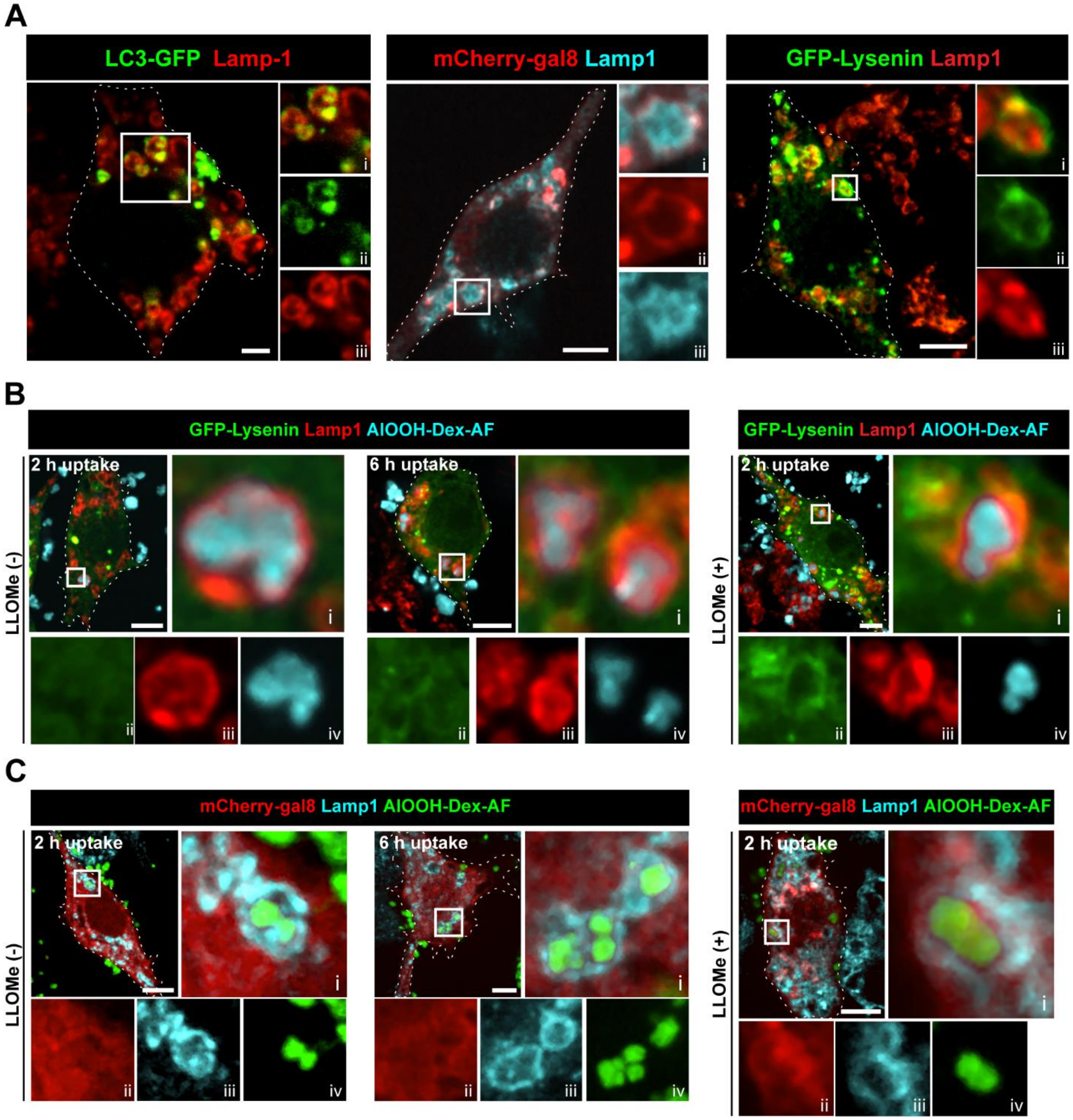

Supplementary Figure 3. Membrane integrity of ACCs. A) RAW cells were transiently transfected with constructs for LC3-GFP, mCherry-galectin8 (mCherry-gal8) and GFP-Lysenin. 14 h post-transfection, cells were treated with the lysomotropic agent LLOMe and then immunostained against Lamp-1. B) (Left panel) RAW cells transiently transfected GFP-Lysenin

with were incubated with AlOOH-AF647 at 37 °C for 2 or 6 h and subsequently processed for immunofluorescence against Lamp-1. (Right panel) After 2 h of incubation with fluorescent adjuvant at 37 °C cells were treated with LLOMe and then processed for immunofluorescence against Lamp-1. C) (Left panel) RAW cells transiently transfected with mCherry-galectin-8 were incubated with AlOOH-Dex-AF488 at 37 °C for 2 or 6 h and subsequently processed for immunofluorescence against Lamp-1. (Right panel) After 2 h of incubation with fluorescent adjuvant at 37 °C, the cells were treated with LLOMe and then processed for immunofluorescence against Lamp-1. B-C) Framed areas are enlarged in (i-iv). Specifically, (i) represents the merge while (ii-iv) represent single fluorescent channels. Spinning disk confocal images correspond to a single z-plane. Images are representative of three independent trials. Over 100 cells were analyzed per trial per condition. Scale bars, 5μm.

Supplementary Figure 4

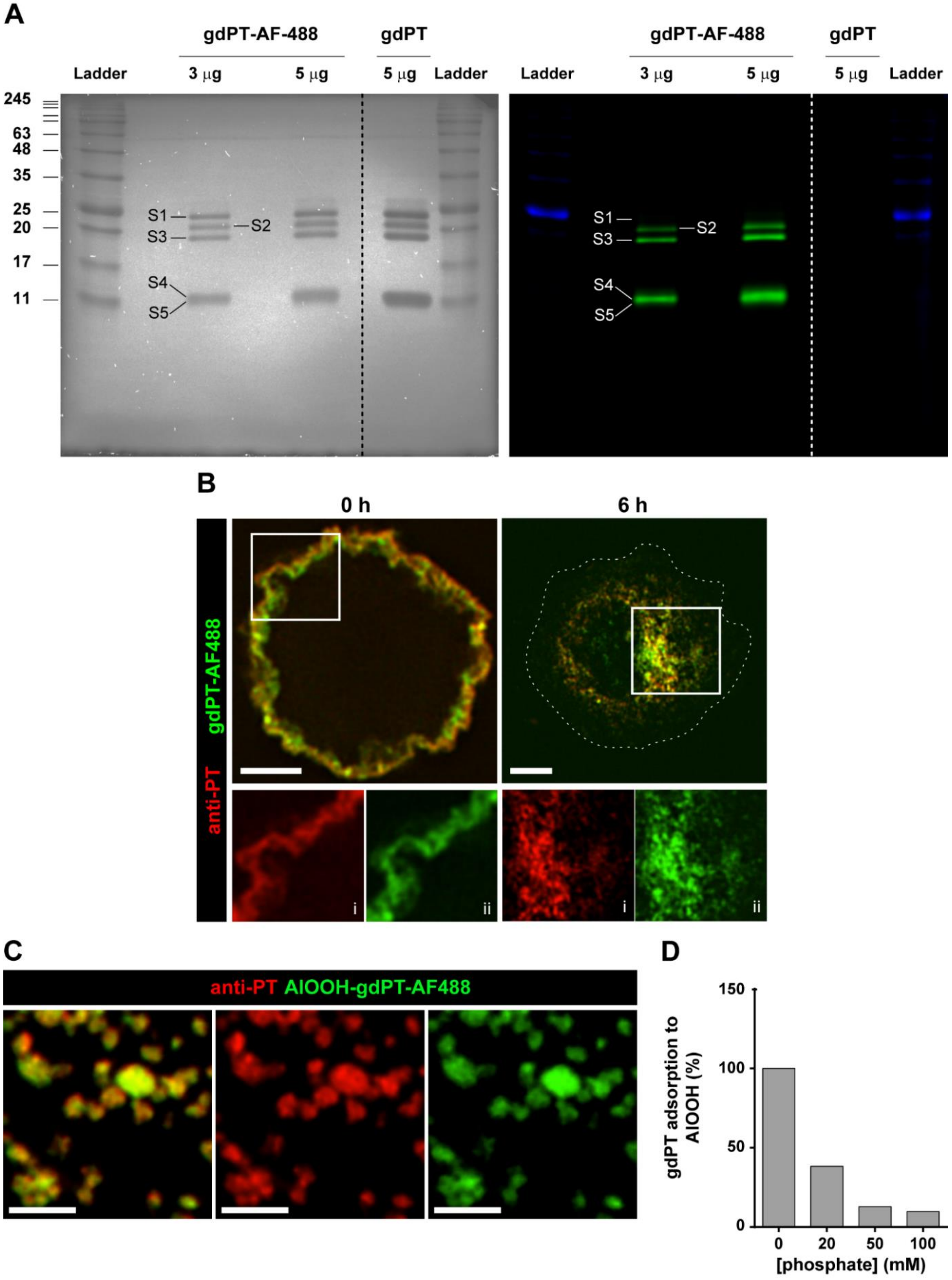

Supplementary Figure 4. gdPT-Alexa Fluor di-conjugates. A) gdPT was conjugated to Alexa Fluor® 488 as described in experimental procedures and processed by SDS-PAGE. Representative image showing PAGE migration of gdPT-AF488 revealed by InstantBlue™ staining (left) and fluorescence (right; green fluorescence at 490 nm). Far-red fluorescence detected at 630 nm (pseudocoloured blue) was used to detect the molecular weight marker (25 kDa). gdPT subunits 1-5 are labelled S1-S5, respectively. B) U-937 cells were incubated with gdPT-AF488 at 37 °C and processed for immunofluorescence with an anti-PT antibody at the indicated time points. (i) and (ii) represent individual fluorescent channels enlarged from the framed area in the merge above. 25 cells were analyzed per trial per condition. C) Fluorescent gdPT was adsorbed to adjuvant particles as described in experimental procedures, and subsequently processed for immunofluorescence as in (B). D) bar graph showing the adsorption percentage of gdPT to AlOOH particles incubated with in the presence of increasing concentrations of sodium phosphate buffer pH 7.4 (see Experimental Procedures). Spinning disk confocal images correspond to a single z-plane. Images are representative of three independent trials. Scale bars, 5μm.

Supplementary Figure 5

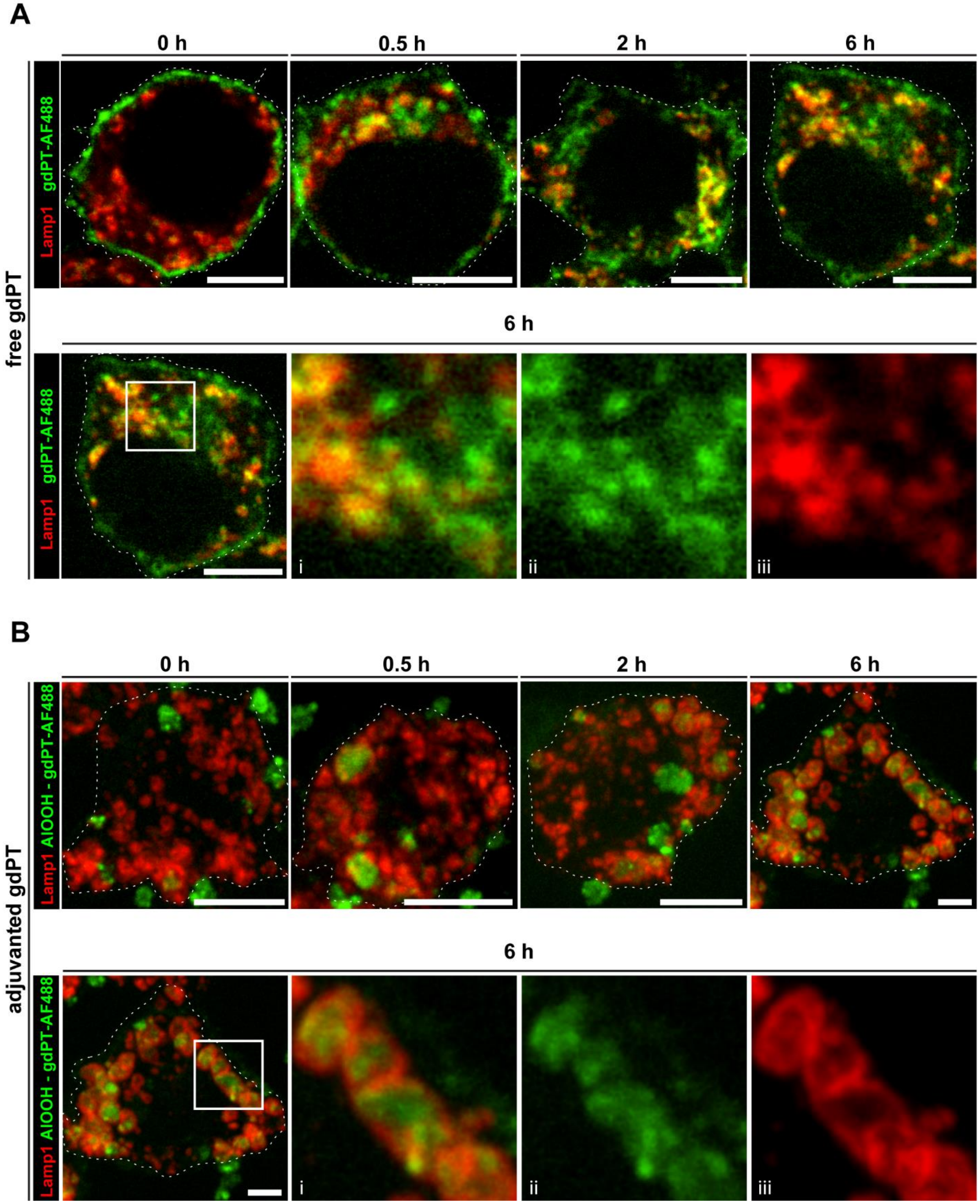

Supplementary Figure 5. Intracellular trafficking of free and adjuvanted fluorescent gdPT in RAW macrophages. (A-B) RAW cells were incubated with gdPT-AF488 or AlOOH-gdPT-AF488 at 37 °C. Subsequently, cells were processed for immunofluorescence against Lamp-1 at the indicated timepoints. Framed areas are enlarged in (i-iii). Specifically, (i) represents the merge while (ii-iii) represent single fluorescent channels. Spinning disk confocal images correspond to a single z-plane. Images are representative of three independent trials. 50 cells were analyzed per trial per condition. Scale bars, 5μm.

Supplementary Figure 6

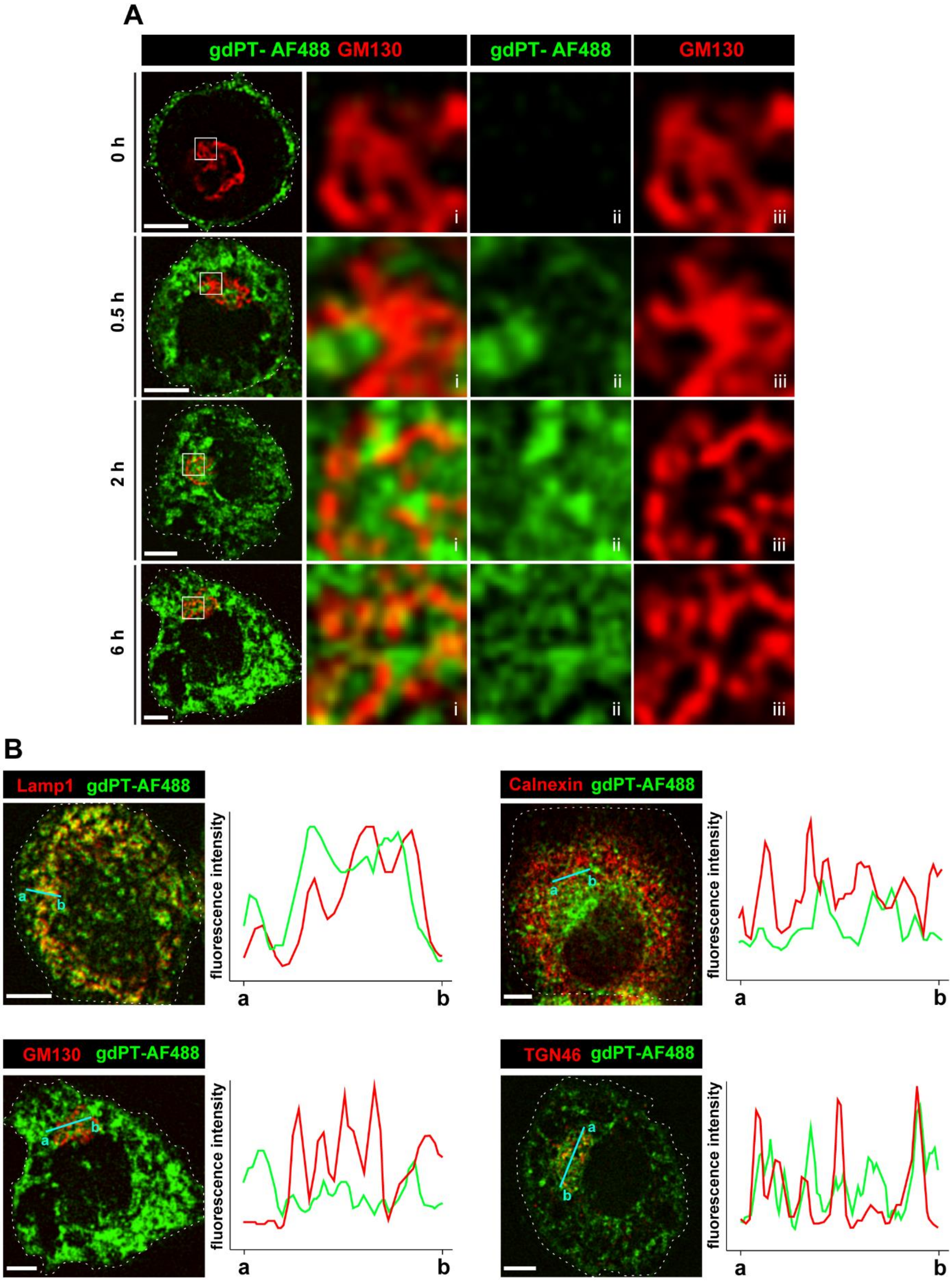

Supplementary Figure 6. Intracellular trafficking of free fluorescent gdPT in U-937 macrophages. A) U-937 macrophages were incubated with gdPT-AF488 and subsequently processed for immunofluorescence against GM130 at the indicated timepoints. Framed areas are enlarged in (i-iii). Specifically, (i) represents the merge while (ii-iii) represent single fluorescent channels. B) Representative micrographs of a 6 h coincubation with gdPT-AF488 immunostained against Lamp-1, calnexin, GM130 and TGN46 accompanied by fluorescence intensity profiles that correspond to the cyan line in each micrograph. Spinning disk confocal images correspond to a single z-plane. Images are representative of three independent trials. 30 cells were analyzed per trial per condition. Scale bars, 5μm.

Supplementary Video Legends

Supplementary Video S1. RAW cells capturing and internalizing AlOOH particles. RAW cells were incubated with AlOOH for 5 min at 37°C prior to live cell imaging. Time-lapse brightfield microscopy series was acquired every 35 s in an Etaluma LS720 Live Cell Microscope under tissue culture conditions. Displayed at a rate of 5 frames s^{-1}. Arrows point to adjuvant particles being captured by filipodia and internalized by macrophages. Videos are representative of three independent trials. 20 cells per trial were analyzed.

Supplementary Video S2. U-937 cells capturing and internalizing AlOOH particles. U-937 cells were incubated with AlOOH for 5 min at 37 °C prior to live cell imaging. Time-lapse brightfield microscopy series was acquired every 35 s in an Etaluma LS720 Live Cell Microscope under tissue culture conditions. Displayed at a rate of 5 frames s^{-1}. Arrows point to adjuvant particles being captured by filipodia and

internalized by macrophages. Videos are representative of three independent trials. 20 cells per trial were analyzed.

Supplementary Video S3. RAW cells capture and internalize fluorescently labeled AlOOH particles. RAW cells stably expressing LifeAct-RFP emit actin enriched protrusions that capture and internalize AlOOH-lumo particles. RAW cells were incubated with AlOOH-lumo for 5 min at 37 °C prior to live cell imaging. Time-lapse confocal microscopy series was acquired every 10 s under tissue culture conditions. Displayed at a rate of 10 frames s^{-1}. Each still corresponds to a confocal section of the cell. Videos are representative of three independent trials. 20 cells per trial were analyzed.

Supplementary Video S4. U-937 cells capture and internalize fluorescently labeled AlOOH particles. U-937 cells were given the SiR-actin probe for 4 h prior to live cell imaging. Cells were incubated with AlOOH-lumo for 5 min at 37 °C prior to live cell imaging. Time-lapse confocal microscopy series was acquired every 10 s under tissue culture conditions. Displayed at a rate of 10 frames s^{-1}. Each still corresponds to a confocal section of the cell. Videos are representative of three independent trials. 20 cells per trial were analyzed.

Supplementary Video S5. RAW cells capture and internalize untreated and IgG-opsonized AlOOH-Dex-AF particles. RAW cells stably expressing LifeAct-RFP were incubated for 5 min at 37 °C with untreated AlOOH-Dex-AF647 and AlOOH-Dex-AF488 previously opsonized with human IgG, simultaneously, for 2 hours at RT. Time-lapse confocal microscopy series acquired every 10 s under tissue culture conditions. Displayed at a rate of 10 frames s^{-1}. Each still corresponds to a confocal section of the cell. Videos are representative of three independent trials. 20 cells per trial were analyzed.

Supplementary Video S6. U-937 cells internalize gdPT-AF. U-937 cells were incubated with gdPT-AF488 at 4 °C for 30 min and time-lapse confocal imaging commenced immediately afterwards under tissue culture conditions. Images acquired every 1 min and displayed at a rate of 5 frames s^{-1}. Each still corresponds to a single z-plane. Videos are representative of three independent trials. 20 cells per trial were analyzed.
